# Engineered CaM2 modulates nuclear calcium oscillation and enhances legume root nodule symbiosis

**DOI:** 10.1073/pnas.2200099119

**Published:** 2022-03-24

**Authors:** Pablo del Cerro, Nicola M. Cook, Rik Huisman, Pierre Dangeville, Lauren E. Grubb, Clemence Marchal, Anson Ho Ching Lam, Myriam Charpentier

**Affiliations:** ^a^Cell and Developmental Biology Department, John Innes Centre, Norwich NR4 7UH, United Kingdom

**Keywords:** root legume symbiosis, calcium signaling, calmodulin

## Abstract

The key physiological event essential to the establishment of nitrogen-fixing bacteria and phosphate-delivering arbuscular mycorrhizal symbioses is the induction of nuclear calcium oscillations that are required for endosymbioses. These regular fluctuations in nucleoplasmic calcium concentrations are generated by ion channels and a pump located at the nuclear envelope, including the CYCLIC NUCLEOTIDE GATED CHANNEL 15 (CNGC15). However, how the CNGC15s are regulated in planta to sustain a calcium oscillatory mechanism remains unknown. Here, we demonstrate that the CNGC15s are regulated by the calcium-bound form of the calmodulin 2 (holo-CaM2), which, upon release of calcium, provides negative feedback to close the CNGC15s. Combining structural and evolutionary analyses of CaM residues with bioinformatic analysis, we engineered a holo-CaM2 with an increased affinity for CNGC15s. In planta, the expression of the engineered holo-CaM2 accelerates the calcium oscillation frequency, early endosymbioses signaling and is sufficient to sustain over time an enhanced root nodule symbiosis but not an increased arbuscular mycorrhization. Together, these results reveal that holo-CaM2 is a component of endosymbiosis signaling required to modulate CNGC15s activity and the downstream root nodule symbiosis pathway.

## Significance

Oscillations in intracellular calcium concentration play an essential role in the regulation of multiple cellular processes. In plants capable of root endosymbiosis with nitrogen-fixing bacteria and/or arbuscular mycorrhizal fungi, nuclear localized calcium oscillations are essential to transduce the microbial signal. Although the ion channels required to generate the nuclear localized calcium oscillations have been identified, their mechanisms of regulation are unknown. Here, we combined proteomics and engineering approaches to demonstrate that the calcium-bound form of the calmodulin 2 (CaM2) associates with CYCLIC NUCLEOTIDE GATED CHANNEL 15 (CNGC15s), closing the channels and providing the negative feedback to sustain the oscillatory mechanism. We further unraveled that the engineered CaM2 accelerates early endosymbioses and enhanced root nodule symbiosis but not arbuscular mycorrhization.

Nutrient acquisition is fundamental to life. Plants have evolved strategies to overcome soil phosphate limitation and gain access to atmospheric dinitrogen by developing beneficial associations with arbuscular mycorrhizal (AM) fungi and nitrogen-fixing bacteria, respectively. Unlike other crops, the vast majority of legumes have mastered associations with both endosymbionts, positioning them as key crops to develop sustainable agricultural practices in both developed and developing countries (1).

The entry of nitrogen-fixing bacteria, known as rhizobia, and AM fungi into legume roots is initiated by the recognition of the endosymbiont. Host plants have plasma-membrane receptor-like kinases (2–6) that recognize rhizobial elicitors, lipochitooligosaccharides (LCOs), also known as Nod factors (7), and mycorrhizal factors composed of derivatives of LCOs and shorter chain chitooligosaccharides (8, 9). Although rhizobial and AM elicitors are recognized by different complexes of receptor-like kinases (10, 11), both symbionts require the activation of calcium oscillations in root epidermal nuclei (9, 12, 13) to set off the endosymbiosis program. In the model legume *Medicago truncatula*, two types of nuclear envelope localized ion channels are required to generate the calcium oscillation; the DOESN’T MAKE INFECTIONS1 (DMI1) channel and paralogs of CYCLIC NUCLEOTIDE GATED CHANNEL 15 (CNGC15) (14), and the calcium pump, MCA8 (15). Similar to the animal CNGCs, plant CNGCs are tetrameric ion channels that can include different CNGC units (16, 17). In *M. truncatula*, CNGC15a, CNGC15b, and CNGC15c are all involved in nuclear calcium oscillation in the root epidermis, nodulation, and arbuscular mycorrhization, suggesting that the three units could assemble into a heterocomplex at the nuclear envelope (14). However, how CNGC15s are regulated in planta to sustain a calcium oscillatory mechanism remains unknown.

In this study, we demonstrate that CNGC15s are regulated by the calcium-bound form of the calmodulin 2 (holo-CaM2) in planta, which shapes the oscillatory pattern of nucleoplasmic calcium concentration by providing negative feedback on CNGC15s to cause its closure. By engineering CaM2 to generate CaM2^R91A^, which specifically increased holo-CaM2 binding affinity to each CNGC15 unit, we accelerated closure of CNGC15s and increased the calcium oscillation frequency. We further show that accelerating the calcium oscillation frequency was sufficient to accelerate the early endosymbiosis signaling and that the expression of CaM2^R91A^ resulted in an enhanced root nodule symbiosis but not enhanced AM colonization. Our data reveal differential regulation of rhizobia and AM endosymbioses by CaM2^R91A^ and suggest that modulating calcium signaling can be used as a strategy to positively impact symbiosis with nitrogen-fixing bacteria.

## Results

### Holo-CaM2 Interacts with CNGC15a, -b, and -c.

**Fig. 1.**
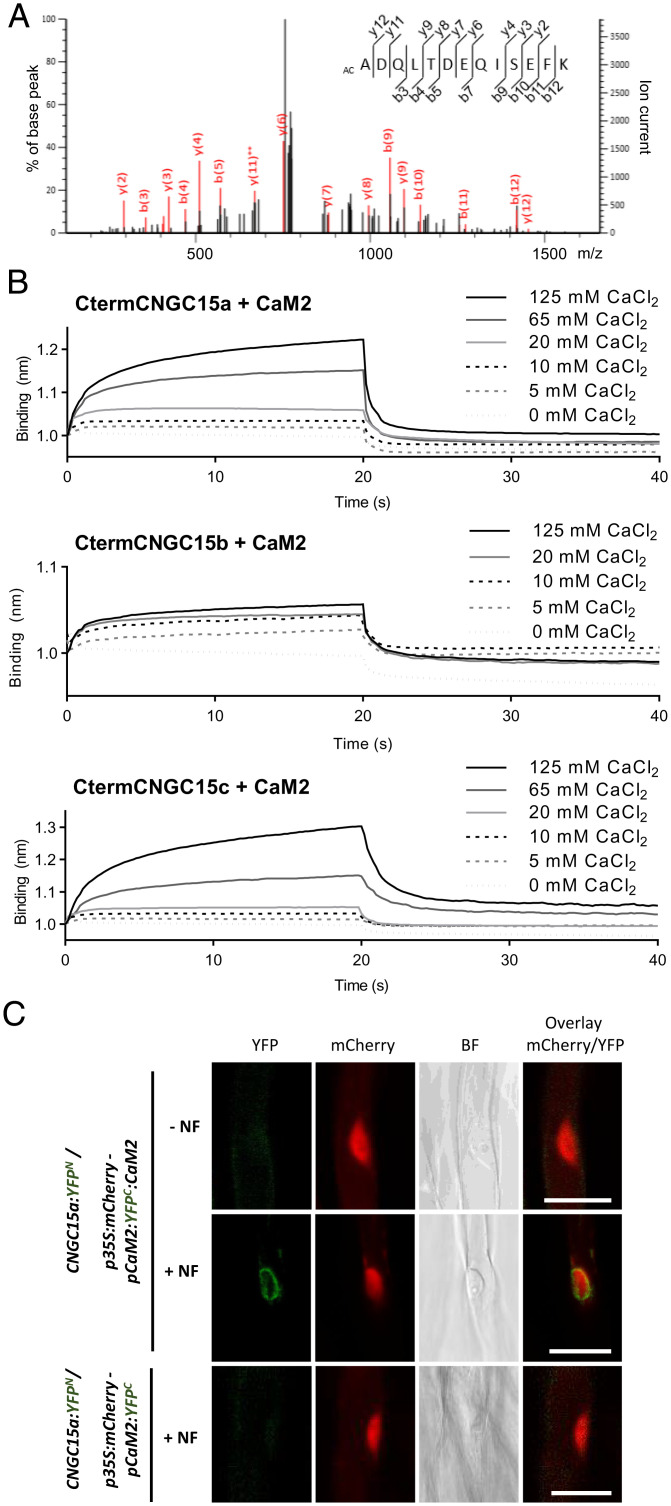
HoloCaM2 interacts with CNGC15a, -b, and -c. (*A*) Representative mass spectra of specific peptide of CaM2, ADQLTDEQISEFK, identified by IP of CNGC15a in *M. truncatula* roots in the presence of calcium, with a probability of 100%. (*B*) Biolayer interferometry analyses of CaM2 binding to CtermCNGC15a, CtermCNGC15b, or CtermCNGC15c. CaM2 binds to CtermCNGC15s specifically in the presence of calcium. The interactions are assessed with 50 µM of CtermCNGC15a, CtermCNGC15b, or CtermCNGC15c and 150 µM of CaM2 in the absence or presence of CaCl_2_ at the concentration indicated. The raw curves represent the average of three replicates normalized to control run and performed using three independent protein purifications. Graphs show the association (0 to 20 s) and dissociation (20 to 40 s) steps of the interaction. (*C*) Bimolecular fluorescence complementation (BiFC) assessed in root hair cells of the *M. truncatula* transgenic line *CNGC15a:Myc:YFP^N^* expressing under the promoter *CaM2 CtermYFP* (*YFP^C^*) fused or not to *CaM2*. The interactions are visualized in the absence (−) or presence (+) of 10^−8^ M of Nod factor (NF) incubated for 1 h. Representative pictures of three biological replicates. (Scale bar, 20 µm.)

To identify regulators of CNGC15s, an *M. truncatula* root complementary DNA library was generated and screened in a yeast two-hybrid assay using the nucleoplasmic C-terminal domain (Cterm) of CNGC15a as a bait. This approach led to the identification of CALMODULIN (CaM)1, 2, and 3 interacting via the highly conserved CaM-binding site (18), an isoleucine-glutamine (IQ)–containing motif, specifically at the CNGC15a-Cterm (*SI Appendix*, Fig. S1*A*). The *M. truncatula* genome comprises four CaMs (*SI Appendix*, Fig. S2 and Dataset S1), and consistent with the identification of *CaM1*, *CaM2*, and *CaM3* via the screening of a root cDNA library, they are all expressed in roots, with the exception of *CaM4* (*SI Appendix*, Fig. S3*A*). To assess whether CaMs interact with CNGC15a in planta, we performed immunoprecipitation (IP) of CNGC15a, followed by mass spectrometry (MS) using transgenic *M. truncatula* plants expressing *CNGC15a* fused to the *Myc* and N-terminal half of the *yellow fluorescent protein* (*YFP^N^*) tags (*SI Appendix*, Fig. S4*A*). The IP was performed in the presence of calcium or ethylenediaminetetraacetic acid (a calcium chelator) to determine the requirement for calcium for copurification of CaMs with CNGC15a in planta. This analysis identified different CaM peptides, among which were peptides specific to CaM2, exclusively in the presence of calcium (Fig. 1*A* and *SI Appendix*, Fig. S4*B*). This suggests that holo-CaM2 interacts with CNGC15a in *M. truncatula* roots. In line with this result, *CaM2* is expressed in root hairs in the absence and presence of symbionts (*SI Appendix*, Fig. S3 *B* and *C*) and localizes to the nucleus as well as to the cytoplasm (*SI Appendix*, Fig. S3*D*). To further test the interaction of holo-CaM2 with the three CNGC15 paralogs, we assessed the capacity of CaM2 to bind the Cterm of CNGC15a, -b, and -c in the presence and absence of calcium using biolayer interferometry (Fig. 1*B*). Consistent with in planta IP-MS, holo-CaM2 bound CtermCNGC15a and similarly interacted with CtermCNGC15b and CtermCNGC15c (Fig. 1*B*). Nod factors–induced nuclear calcium oscillation raises the nucleoplasmic calcium level above 700 nM in root hair of *M. truncatula* after 10 to 20 min of treatment (13) To test whether Nod factor promotes holo-CaM2 interaction with CNGC15a, we performed bimolecular fluorescence complementation analyses by expressing *CaM2* fused to *YFP^N^* under its native promoter in the transgenic *M. truncatula* roots expressing *CNGC15a:Myc:YFP^N^.* After 1 h of incubation with or without 10^−8^ M Nod factor of *Sinorhizobium meliloti*, we observed specific interaction of CaM2 and CNGC15 at the nuclear envelope of root hair cells of the induction zone, exclusively in the presence of Nod factor (Fig. 1*C*). Altogether, our results demonstrate that the calcium-bound form of CaM2 interacts with the Cterm of the CNGC15s and that this interaction is promoted in root hair cells by Nod factor, which induces nuclear calcium oscillations.

### CaM2^R91A^ Has an Increased Binding Affinity for CNGC15a, -b, and -c.

**Fig. 2.**
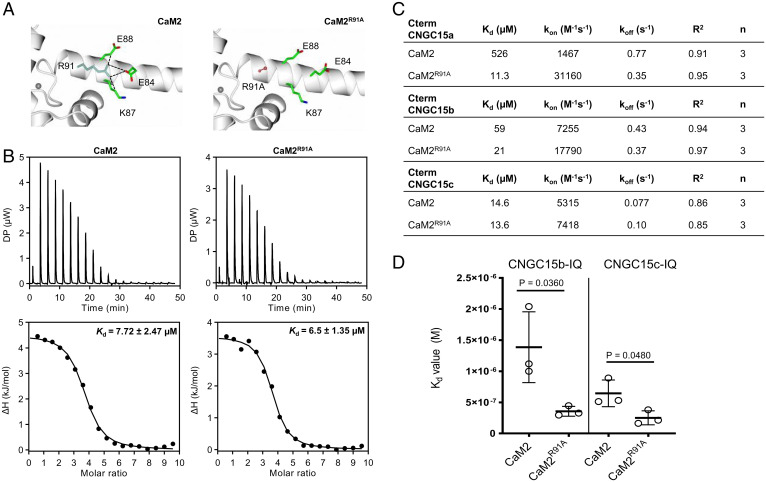
Mutation R91A increases holoCaM2 binding affinity and association rate for CNGC15a, CNGC15b, and CNGC15c. (*A*) Structure homology models of CaM2 and CaM2^R91A^. The R91A mutation abolishes contacts with the side chains of E86, K87, and E83, causing E83 to form a different orientation. The R91 residue is shown in blue and mutated R91A in red, with amino acid contacts shown in green. Contacts between amino acid side chains are shown by black dashed lines. (*B*) Isothermal titration calorimetry of CaM2 (100 µM) and CaM2^R91A^ (100 µM) with 5 mM CaCl_2_. *Top* shows representative thermogram obtained for automatic injections of CaCl_2_ over time. Differential power, DP. *Bottom* presents the integrated curve of the experimental points (black circle); K_d_, average dissociation constant from five replicates using protein from two independent purifications. Enthalpy of interaction, ΔH. Student’s *t* test. No statistical differences were observed for CaM2 and CaM2^R91A^. (*C*) Kinetic parameters measured via Biolayer interferometry in the presence of 20 mM CaCl_2_. Data are determined by the global fit of the interaction with 25 µM, 50 µM, and 100 µM of CaMs and 20 µM of His6-MBP:CtermCNGC15s. k_on_ (M^−1^s^−1^), association rate constant; k_off_ (s^−1^), dissociation rate constant; R^2^, coefficient of determination; *n*, number of replicates performed using one protein purification. (*D*) K_d_ of CaM2 and CaM2^R91A^ with 10 µM of CNGC15b-IQ peptide (AACFIQVAWRRTIQEKKG) or CNGC15c-IQ peptide (AACFIQAAWRRHKKRKEA) measured via isothermal titration calorimetry in the presence of 5 mM CaCl_2_. Three independent replicates were performed using proteins from two independent purifications. Error bars represent SD. Student’s *t* test. *P* < 0.05.

CaMs are present in all eukaryotes and are highly conserved throughout evolution (19). In humans, despite the presence of three *CaM* genes encoding identical proteins, several amino acid substitutions have been linked to specific phenotypes associated with the misregulation of ion channels (20, 21). Notably, CaM mutations specifically impair the regulation of the ryanodine receptor RyR2 (21). This observation shows that despite the myriad of processes involving calmodulin, mutations in CaM can lead to specific effects associated with a specific target. The calmodulin EF-hands are structurally organized in globular pairs forming the N- and C-terminal lobes, which are connected by the interlobe linker. Upon calcium binding to the EF-hands, the interlobe linker contributes to conformational changes of the calmodulin, presenting a structurally dynamic surface that binds its target proteins (22, 23). Studies have led to the hypothesis that the flexibility provided by this region is essential for CaM‐target binding affinity (24). To investigate how holo-CaM2 contributes to CNGC15s regulation, we challenged this hypothesis by engineering the interlobe linker of CaM2. As such, we aimed to increase CaM2 binding affinity for CNGC15s without disrupting the calcium affinity of CaM2, as calcium is required for CaM2 to bind CNGC15s. Halling and coworkers (19) previously analyzed the sequence structural conservation in CaM at each residue position across eukaryotes. This study highlights the substitutions that are tolerated in different phyla and the residues important for the target binding. Based on this analysis, we focused on amino acids of the interlobe linker, which have a high frequency of alternative residues and are not in contact with the target protein (19). We hypothesized that mutations in these residues could increase the flexibility of CaM2 and thus increase its binding affinity for CNGC15 without altering calcium binding and the target recognition. The five residues facing the solvent with a low frequency of conservation were selected (19), as shown in *SI Appendix*, Table S1. We submitted the corresponding CaM2 mutant variants where each selected residue is substituted to an alanine to DynaMut (25) and assessed the effect of each mutation on CaM2 dynamics (*SI Appendix*, Table S1). The R91A substitution was predicted to generate the highest increase in CaM2 flexibility by abolishing contacts with the side chains E86, K87, and E83, (Fig. 2*A* and *SI Appendix*, Table S1 and Fig. S5). To assess the effect of R91A on CaM2 calcium binding affinity, we conducted isothermal titration calorimetry analysis with CaM2 and CaM2^R91A^ in the presence of 5 mM CaCl_2_. The dissociation constant (K_d_) values and the thermodynamic parameters for CaM2 and CaM2^R91A^ were similar, demonstrating that R91A does not affect calcium binding affinity (Fig. 2*B* and *SI Appendix*, Fig. S6). To evaluate the effect of R91A on holo-CaM2 interaction with CNGC15s, we performed biolayer interferometry using the Cterm-CNGC15s (Fig. 2*C* and *SI Appendix*, Fig. S7). The K_d_ values for holo-CaM2^R91A^ to CtermCNGC15a and CtermCNGC15b were strongly lower than for holo-CaM2 (Fig. 2*C*). In contrast, the K_d_ values for holo-CaM2^R91A^ to CtermCNGC15c were only slightly reduced, although the association rate constant of holo-CaM2^R91A^ was higher than that of holo-CaM2 for the three Cterm-CNGC15s (Fig. 2*C*). To determine whether holo-CaM2^R91A^ has a higher affinity for the Cterm-CNGC15c with a more sensitive method, we performed isothermal titration calorimetry measurements using peptides corresponding to the IQ motif of CNGC15c as well as to the IQ motif of CNGC15b (Fig. 2*D* and *SI Appendix*, Fig. S8). The binding analyses revealed that the K_d_ value for holo-CaM2^R91A^ to the IQ motif of CNGC15c was significantly lower than holo-CaM2 and confirmed that holo-CaM2^R91A^ has a higher affinity for CNGC15b (Fig. 2*D*). Altogether, our results demonstrate that R91A had no effect on calcium binding to holo-CaM2 but was sufficient to increase the binding affinity and the association rate of holo-CaM2 to CNGC15a, CNGC15b, and CNGC15c.

### CaM2^R91A^ Closes CNGC15s and Accelerates the Nuclear Calcium Oscillation Frequency.

**Fig. 3.**
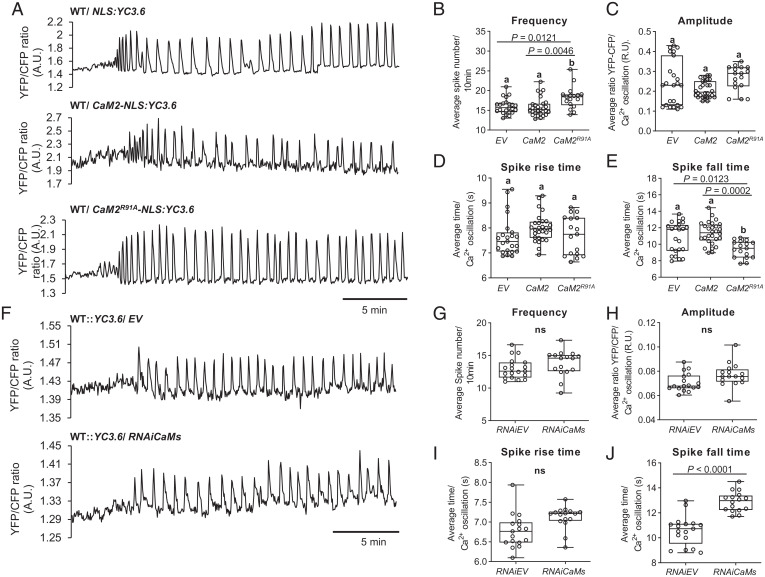
CaM2^R91A^ increases nuclear calcium oscillation frequency. (*A*) Representative Nod factor (10^−8^ M) induced calcium oscillation in *M. truncatula* WT root hair cells expressing the nuclear localized yellow cameleon 3.6 (*NLS:YC3.6*) or coexpressing *NLS:YC3.6* with *CaM2* or *CaM2^R91A^* driven by the *L. japonicus* ubiquitin promoter. The traces show the ratio of YFP/CFP fluorescence in arbitrary units (A.U.). (*B*–*E*) Analyses of the frequency (*B*), the amplitude (*C*), the duration of the spike’s upward slope (rise time) (*D*), and the duration of the spike’s downward slope (fall time) (*E*) of the nuclear calcium oscillations recorded in *A* with *NLS:YC3.6* (*n* = 23), *CaM2-NLS:YC3.6* (*n* = 28), and *CaM2^R91A^-NLS:YC3.6* (*n* = 17). Different letters indicate different statistical groups (one-way ANOVA; post hoc Bonferroni). (*F*) Representative Nod factor (10^−8^ M) induced calcium oscillation in *M. truncatula* WT::*YC3.6* root hair cells expressing *CaM2* hairpin construct (*RNAiCAMs*) or the empty vector (EV). (*G*–*J*) Analyses of the frequency (*G*), the amplitude (*H*), the duration of the spike’s upward slope (*I*), and the duration of the spike’s downward slope (*J*) of the nuclear calcium oscillations recorded in *F* with *EV* (*n* = 17), *RNAiCaMs* (*n* = 16). Student’s *t* test. *P* ≤ 0.0001. (*B*–*J*) Error bars represent SD. YFP, yellow fluorescent protein; CFP, cyan fluorescent protein; ns, no significant difference. R.U., relative units.

In response to symbiotic factors, nuclear calcium oscillations are generated by the interplay of two ion channels, DMI1 and CNGC15s, and a type IIA calcium ATPase, MCA8 (26). Similar to its closest *Arabidopsis thaliana* homologs, AtECA1 and AtECA4, MCA8 does not possess any calmodulin-binding regulatory domain [*SI Appendix*, Fig. S9 (26, 27)], and DMI1 does not interact with any of the three CaMs or CaM2^R91A^ in pairwise yeast two hybrids (*SI Appendix*, Fig. S10). As holo-CaM2 binds CNGC15s, we explored the role of holo-CaM2 in regulating CNGC15s and modulating nuclear calcium oscillations using the engineered CaM2^R91A^. In planta, CaM2^R91A^ with an increased binding affinity and association rate to CNGC15s will outcompete endogenous CaM2. Thus, we predicted that CaM2^R91A^ would amplify the role of CaM2 in regulating CNGC15s. As holo-CaM2 binds to CNGC15s, we hypothesized three scenarios by which holo-CaM2 might modulate CNGC15s activity (*SI Appendix*, Fig. S11): holo-CaM2 could close the CNGC15s after each calcium release (*SI Appendix*, Fig. S11*B*), reopen CNGC15s after the first calcium spike (*SI Appendix*, Fig. S11*C*), or alternatively terminate prematurely the calcium oscillation (*SI Appendix*, Fig. S11*D*). Using *Agrobacterium rhizogenes*–mediated transformation, we generated *M. truncatula* wild-type (WT) roots co-overexpressing *CaM2* or *CaM2^R91A^* driven by the *Lotus japonicus UBIQUITIN* promoter and the Förster resonance energy transfer–based calcium reporter yellow cameleon 3.6 fused to a nuclear localization signal (*NLS:YC3.6*). After treatment with Nod factor, nuclear calcium oscillations were recorded for at least 40 min, a duration that is sufficient to produce a minimum number of 36 calcium spikes required for downstream transcriptional activation of symbiosis genes (28). The calcium oscillations recorded were subsequently analyzed for each parameter defining the oscillations, which included the amplitude, duration, and frequency (*SI Appendix*, Fig. S11*A* and Fig. 3 *A*–*E*). We observed that overexpressing *CaM2^R91A^* did not prematurely terminate the calcium oscillation but significantly increased the frequency of the oscillation without affecting its amplitude in comparison to the root expressing the empty vector or overexpressing *CaM2* (Fig. 3 *B* and *C*). Analysis of the duration of the upward and downward slopes of each spike revealed that the acceleration of the calcium oscillation frequency was associated with a decrease in the duration of the downward slope of the calcium spikes (Fig. 3 *D* and *E*), suggesting that holo-CaM2 is required to close CNGC15s after each calcium release.

To confirm this result, we generated an RNA interference construct knocking down *CaM2*. As the three *CaMs* are highly conserved, this construct is also a knockdown of *CaM1* and *CaM3* and is therefore called *RNAiCaMs* (*SI Appendix*, Fig. S12). Consistent with the expression of *CaM2^R91A^*, the analysis of the calcium oscillation induced upon Nod factor treatment in the root expressing *RNAiCaMs* revealed that silencing *CaMs*, including *CaM2*, specifically increased the duration of the downward slope of each calcium spike (Fig. 3 *A* and *F*–*J*). This result indicates that knocking down *CaM2* interfered with the closure of CNGC15s, and in line with the gain of function of CaM2^R91A^, this result confirmed that CaM2 is required to close CNGC15s. Interestingly, silencing *CaM2* did not significantly decrease the frequency of the calcium oscillation (Fig. 3*G*), demonstrating that the time to reactivate the calcium release was reduced. This observation suggests that the resting time between each calcium release was influenced by CaM2. In line with the role of CaM2 in the closure of CNGC15s, the dissociation of CaM2 from CNGC15s would be required to reopen the CNGC15s. Hence, the reduced amount of CaM2 could favor reopening of the ion channels and thus reduce the resting time.

### CaM2^R91A^ Enhances Root Nodule Symbiosis.

**Fig. 4.**
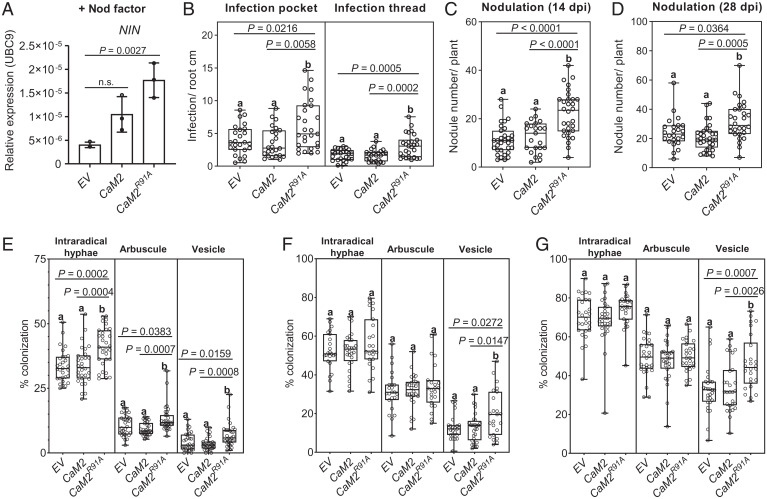
CaM2^R91A^ enhances root nodule symbiosis. (*A*–*G*) *M. truncatula* roots overexpressing *CaM2*, *CaM2^R91A^,* or the empty vector (*EV*) were assessed for *NIN* expression in response to Nod factor (*A*), endosymbioses association with *S. meliloti* 2011 (*B*–*D*), and arbuscular mycorrhiza (*E*–*G*). (*A*) Expression analyses of the endosymbiosis-induced gene *NIN* by qRT-PCR in hairy roots expressing the indicated genetic construct. Hairy roots were harvested 6 h after treatment with 10^−8^ M Nod factor. Expression was normalized to *UBC9* (TC106312). Bars, error bars, and circles represent mean, SD, and individual values, respectively (*n* = 3). *P* values (*P*) are shown (Dunnett’s test, comparison to *EV*). n.s., no significant difference. (*B*) Number of infection pockets and infection threads per root length at 6 d post inoculation (dpi) with *Sm*2011. (*C* and *D*) Number of nodules per plant after 14 dpi (*C*) and 28 dpi (*D*) with *Sm*2011. (*E*–*G*) Percentage of root length colonization of *R. irregularis* including intraradical hyphae, arbuscule, and vesicle at early stage of colonization (*E*), mid stage of colonization (*F*), and later stage of colonization (*G*). Early, mid, and late stages of colonization correspond to 20, 36, and 50 d post inoculation (dpi), respectively. Results represent the average of three biological replicates. Different letters indicate significant differences (one-way ANOVA; post hoc Bonferroni). (*B*) *n* (*EV*) = 25, *n* (*CaM2*) = 24, *n* (*CaM2^R91A^*) = 27. (*C*) *n* (*EV*) = 31, *n* (*CaM2*) = 23, *n* (*CaM2^R91A^*) = 30. (*D*) *n* (*EV*) = 24, *n* (*CaM2*) = 31, *n* (*CaM2^R91A^*) = 28. (*E*) *n* (*EV*) = 30, *n* (*CaM2*) = 30, *n* (*CaM2^R91A^*) = 31. (*F*) *n* (*EV*) = 23, *n* (*CaM2*) = 28, *n* (*CaM2^R91A^*) = 23. (*G*) *n* (*EV*) = 28, *n* (*CaM2*) = 28, *n* (*CaM2^R91A^*) = 26. (*B*–*G*) Error bars represent SD.

Previous studies have demonstrated that reducing the calcium spiking frequency delays the induction of a nodulation marker gene (28). As CaM2^R91A^ accelerated the frequency of the calcium oscillation (Fig. 3), we hypothesized that the expression of nodulation genes such as *NODULE INCEPTION* (*NIN*) (29) would be enhanced in roots overexpressing *CaM2^R91A^*, which could subsequently accelerate the early colonization by the endosymbionts. To test this, we first incubated *M. truncatula* roots overexpressing *CaM2* or *CaM2^R91A^* with 10^−8^ M Nod factor. After 6 h of Nod factor induction, the expression of *NIN* was significantly up-regulated in the roots overexpressing *CaM2^R91A^* and not in the roots overexpressing *CaM2* (Fig. 4*A* and *SI Appendix*, Fig. S13). To assess the early colonization by the endosymbionts, we inoculated *M. truncatula* roots expressing *CaM2* or *CaM2^R91A^* with *Sm*2011 or *Rhizophagus irregularis*. The first colonization step by rhizobia is characterized by the entrapment of the bacteria in curled root hairs forming the so-called infection pockets, from which an infection thread guides the dividing bacteria to the concomitantly developing root nodule. After 6 d of inoculation with *Sm*2011, roots overexpressing *CaM2^R91A^* presented a significant increase in infection pockets and infection threads (Fig. 4*B*). On the other hand, early AM colonization can be monitored over time by assessing the number of fungal structures within the roots, including intraradical hyphae, arbuscules, and vesicles. AM colonization was significantly increased in the roots overexpressing *CaM2^R91A^* after 20 d of inoculation (Fig. 4*E*). Altogether, these results indicate that CaM2^R91A^ is sufficient to increase early nodulation gene expression and early colonization by both endosymbionts, suggesting that the increase in calcium spiking frequency positively regulates early signaling.

**Fig. 5.**
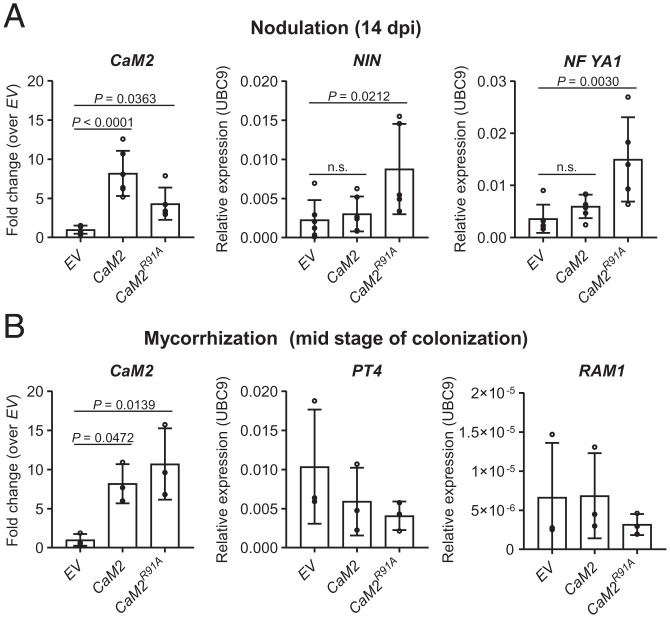
CaM2^R91A^ amplifies transcriptional induction of nodulation genes. (*A* and *B*) Expression analyses of *CaM2* and endosymbiosis-induced genes by qRT-PCR in hairy roots expressing the indicated genetic construct. Hairy roots were harvested 14 d post inoculation (dpi) with *S. meliloti* strain 2011 (*A*) or 36 d after inoculation (mid stage of colonization) with *R. irregularis* (*B*). Expression was normalized to *UBC9* (TC106312). *CaM2* expression is relative to *EV*. Bars, error bars, and circles represent mean, SD, and individual values, respectively. (*A*) *n* (*EV*) = 6, *n* (*CaM2*) = 6, *n* (*CaM2^R91A^*) = 5. (*B*) *n* = 3. *P* values (*P*) are shown (Dunnett’s test, comparison to *EV*). No statistical differences were observed for *PT4* and *RAM1* expressions in the mycorrhization assay. n.s., no significant difference.

To assess whether this increase in colonization by endosymbionts could be sustained over time, we monitored nodulation and AM colonization at later time points. At 14 and 28 d after inoculation with *Sm*2011, overexpressing *CaM2^R91A^* significantly increased the number of nodules in comparison to the empty vector and *CaM2* overexpression (Fig. 4 *C* and *D*), without affecting root growth (*SI Appendix*, Fig. S14). In line with this, nodulation marker genes, including *NIN* and *NF-YA1* (30), were significantly up-regulated in roots overexpressing *CaM2^R91A^* in comparison to the WT after 14-d inoculation with *Sm*2011 (Fig. 5*A*). In contrast, the increase in AM intraradical hyphae and arbuscule formation was not sustained over time, apart from that of AM vesicle formation (Fig. 4 *F* and *G*). The expression of the AM marker gene, *REQUIRED FOR ARBUSCULAR MYCORRHIZATION (RAM1*) (31), was also not significantly up-regulated in roots overexpressing *CaM2^R91A^* at mid stage of AM colonization (Fig. 5*B*). These results demonstrate that in addition to early nodulation signaling, CaM2^R91A^ is sufficient to sustain an increase in root nodule symbiosis, but not AM intraradical hyphae and arbuscule development.

## Discussion

In recent years, CaMs have been highlighted as essential regulators of plant CNGCs (32). However, despite their high sequence conservation across kingdoms, CaMs regulate CNGCs in different ways, including activation and/or inactivation of CNGCs via the calcium-free and/or calcium-loaded form of CaMs (32). This observation suggests that the specificity of the regulation of CNGC complexes by CaMs depends on the CaM isoform and on the nature of the CNGC complex in planta. In this study, we demonstrated that in *M. truncatula* roots, the nuclear localized CNGC15s are regulated by the holo-CaM2. Combining the structural and evolutionary analyses of CaM residues (19) with bioinformatic analysis, we identified the mutation R91A in the interlobe linker as a candidate to increase holo-CaM2 flexibility. We confirmed via biochemical analyses that the predicted increase in flexibility of CaM2^R91A^ is translated into an enhanced binding affinity and association rate to each CNGC15 unit. In roots, the overexpression of CaM2^R91A^ and the silencing of CaM2 revealed that holo-CaM2 closes CNGC15s once calcium is released. This study reveals that engineering CaMs with an increased affinity for their target is a powerful strategy to unravel the role of CaMs in planta. It further positions CaM2 as a player contributing to the negative feedback essential in shaping the oscillatory behavior of the nucleoplasmic calcium concentration. This result is consistent with our previous mathematical model, which predicted negative holo-CaM feedback on CNGC15 activity to sustain the oscillatory mechanism (14). The association of the holo-CaM2 to CNGC15s is thus required to close the channels, and the dissociation of calcium from CaM2 will abolish the holo-CaM2–CNGC15s complex, and thus the negative feedback. The time to release the negative feedback would cause the delay observed between each calcium spike. Combining the role of holo-CaM2 and the mathematical model, we propose that upon activation of either DMI1 or CNGC15, both ion channels undergo a structural change. Calcium leakage through CNGC15 is predicted to positively feed back on DMI1 (33), increasing its counter balance flux and, by consequence, the calcium release via CNGC15. Holo-CaM2 binds CNGC15 and closes CNGC15s, whereas MCA8 pumps calcium back into the nuclear envelope lumen. In this scenario, holo-CaM2 binding is transient, and once holo-CaM2 is released from CNGC15, the cycle repeats itself, as both ion channels are in an active state (*SI Appendix*, Fig. S15). Future work will be required to fully demonstrate this model and to unravel how DMI1 and/or CNGC15 is activated.

In root nodule and AM symbioses, the nuclear calcium oscillation is known to activate at least one calcium decoder, the calcium- and calmodulin-dependent kinase CCaMK, which subsequently activates endosymbioses’ gene expression (34). CCaMK is inactive at basal calcium concentration and requires a high input of calcium, provided by sufficient calcium oscillation, to be turned on via CaM binding (34). However, previous work demonstrated that increasing CCaMK affinity for CaMs does not increase root nodule symbiosis but, on the contrary, impairs nodule development (35). In roots overexpressing CaM2^R91A^, we enhanced the expression of a nodulation marker gene and accelerated the early colonization by both AM and rhizobia. Our results suggest that the positive effect on early root endosymbioses might hypothetically be caused by an accelerated activation of CCaMK, which would be provided by the increased calcium oscillation frequency in roots expressing CaM2^R91A^.

In addition to early signaling, CaM2^R91A^ expression is sufficient to sustain an increase in root nodule symbiosis but not in arbuscule formation, indicating that the calcium signaling networks controlling AM and root nodule symbioses differ. This observation could be explained by alternative specific pathways induced by rhizobia or AM colonization. On one hand, a high frequency of calcium oscillation and/or CaM2^R91A^ could regulate target proteins whose expression is specifically induced by rhizobia and whose activity enhances root nodule symbiosis. On the other hand, AM cortical infection could induce different signaling components insensitive to CaM2^R91A^-dependent calcium signaling changes.

Altogether, our work reveals that calmodulins can be engineered to study their function directly in planta and that modulating calcium signaling could serve as a valuable strategy to enhance nitrogen-fixing bacteria symbiosis. Future investigation will help to understand the underlying mechanisms associated with the enhancement of root nodule symbiosis.

## Materials and Methods

Experiment materials and methods are described in *SI Appendix*, including the full details of the yeast two-hybrid screens, the pairwise yeast two hybrids, the phylogeny, the gene expression analyses, the molecular cloning and plant material, the histochemical GUS staining and subcellular localization, the IP and liquid chromatography–MS/MS assay, the bimolecular fluorescence complementation, the protein expression and purification, the biolayer interferometry, the isothermal titration calorimetry, the structural homology modeling, and the calcium imaging and root endosymbioses analyses.

### Statistical Analysis.

Statistical significance was determined by Student’s *t* test, one-way analysis of variance (ANOVA) followed by post hoc Bonferroni or Dunnett’s test using GraphPad Prism version 8, as indicated.

## Supplementary Material

Supplementary File

Supplementary File

Supplementary File

## Data Availability

Accession numbers of the sequences from this study are listed in Dataset S2 and are available at Phytozome (https://phytozome-next.jgi.doe.gov/). All additional study data are included in the article and/or supporting information.
