# Supplementary material for: Engineered CaM2 modulates nuclear calcium oscillation and enhances legume root nodule symbiosis
Source: Proc Natl Acad Sci U S A. 2022 Mar 24;119(13):e2200099119. doi: 10.1073/pnas.2200099119 (PMC9060481; doi:10.1073/pnas.2200099119)
Supplement: Supplementary File [file pnas.2200099119.sapp.pdf]

PNAS
www.pnas.org

Supplementary Information for

Engineered CaM2 modulates nuclear calcium oscillation and enhances legume root nodule symbiosis

Pablo del Cerro[a,1], Nicola M. Cook[a,1], Rik Huisman[a,b], Pierre Dangeville[a], Lauren E. Grubb[a], Clemence Marchal[a,c], Anson Ho Ching Lam[a], and Myriam Charpentier[a,*]

* corresponding author: Myriam Charpentier
Email: myriam.charpentier@jic.ac.uk

This PDF file includes:

Supplementary Material and methods
Figures S1 to S17
Table S1
Legends for Datasets S1, S2, S3
SI References

Other supplementary materials for this manuscript include the following:

Dataset S1
Dataset S2
Dataset S3

Materials and Methods

Yeast two-hybrid screens. The yeast two-hybrid screen was performed by Hybrigenics Services (http://www.hybrigenics.com, Paris, France) using the company's ULTImate Y2H service. *Medicago truncatula CNGC15a-Cterm* cDNA was subcloned into pB66 as a C-terminal fusion to Gal4 DNA-binding domain (N-Gal4-CNGC15aCterm-C). This bait fragment includes MtCNGC15a (T394 to D710) and was used to screen a random-primed two days old *Medicago truncatula* root infection zone cDNA library constructed into pB27 as a C-terminal fusion to LexA. 66.4 million clones were screened using the mating approach with Y187 and CG1945 (mat a) yeast strains as previously described (1)

Pairwise yeast two hybrids. The Gal4-based yeast two-hybrid assay was performed in the yeast strain AH109 using the lithium acetate method for transformation (2). The C-terminal domain of CNGC15a (T394 to D710), CNGC15a$^{\Delta IQ}$ (T394 to D708), DMI1 (S343 to E882), and the N-terminal domain of CNGC15a (M1 to K84), CNGC15b (M1 to W70), CNGC15c (M1 to K90) and DMI1 (M1 to S128), and the nucleoplasmic loop1 (I139 to E173) and loop2 (S227 to Y247) of CNGC15a were fused to the Gal4 binding domain (BD). The full length CaM2, CaM1, CaM3 and CaM2^{R91A} were fused to the Gal4 activating domain (AD). The murine p53 and its interacting partner the SV40 large T-antigen were used as control (CLONTECH PT3024-1). The vector pBD-GAL4-GW and pAD-GAL4-GW (CLONTECH) were used to clone C-termini or N-termini coding sequences via BP/LR gateway cloning (Invitrogen). The CaM2^{R91A} and CNGC15a$^{\Delta IQ}$ were generated via site directed mutagenesis (3) using the primers described in Dataset S2. CNGC15a$^{\Delta IQ}$ was generated by removing the CaM binding sequence 613-AACFIQAAWFRYKRMKE-629. Analyses of protein expression were monitored by immunoblot detection using the mouse GAL4 DNA-BD Monoclonal Antibody (Takara, dilution 1/4,000) and bovine anti-mouse IgG-HRP (Santa Cruz Biotechnology, dilution 1/10,000).

Phylogeny. Members of the Calmodulin family were identified through BLASTp and BLASTn searches against genomes on Phytozome (4) (Dataset S1). Phylogenetic trees were constructed using the amino acid sequences, and Calmodulin-like proteins were removed from subsequent analyses. Incorrectly predicted gene models were manually curated based on conserved exon-intron structures. DNA coding sequences were then aligned with MAFFT L-INS-i with default settings (5). IQ-Tree (v1.6.12) was used to construct phylogenetic trees with the maximum likelihood approach (6). ModelFinder was used to find the best-fit evolutionary model (TPM2u+R5), and ultrafast bootstrap (UFBoot2) with 1,000 replications was used to estimate branch support (7). Phylogenetic trees were visualized in the interactive Tree Of Life (iTOL) v4 (8).

Gene expression analyses. RNA was extracted from the wild type R108 root induction zone with RNeasy plant mini-Kit (Qiagen) and subsequently treated with TURBO DNA-FREE (Ambion) before performing the reverse transcription with 1 µg RNA using SuperscriptII reverse transcriptase (Invitrogen). The quantitative gene expression was monitored with SYBR® Green based qPCR on a Biorad thermocycler using gene specific primers. *UBC9* (*TC106312*) (9) was used for normalization. Primers used for quantitative RT-PCR are listed in Dataset S2.

Plant material. The stable R108/*p35S:CNGC15a:Myc:YFP^{N}:T35S* line was generated using *Agrobacterium tumefaciens* AGL1 carrying the recombinant binary vector *pB7WG2::CNGC15a:MYC:YFP^{N}* according to (10) with the following modifications. AGL1 was used at OD_{600}=0.6 and no acetosyringone was added. Incubation time with AGL1 was 45 min. Leaf explants were prepared by cutting perpendicular to the main vein. Co-cultivation of leaf explants was carried out with their abaxial surface in contact with the medium. The coding region of *CNGC15a* in frame with the *MYC:YFP^{N}* was generated by overlapping PCR using the primers listed in the Dataset S2 as described previously (3). *MYC:YFP^{N}* was amplified from pDEST-GW SCYNE (11). The sequence *CNGC15a:MYC:YFP^{N}* was cloned into the pB7WG2 (12) via BP/LR gateway cloning.

Stable transformed roots were generated via *Agrobacterium rhizogenes*-mediated gene transfer as previously described (13) using the *A. rhizogenes* strain AR1193.

Histochemical GUS staining and subcellular localization. The native promoter of *CaM2* (2866 bp upstream of the start codon) was amplified from genomic A17 DNA using primer P39/P40 (Dataset S2), and cloned into pKGWFS7-RR containing a β-glucuronidase (GUS) fusion reporter as well as *pAtUBQ10::DsRed1* as plant selectable marker (12) via BP/LR gateway cloning. *Medicago truncatula* A17 roots stably transformed with *pK::pCaM2:GUS-RR* were selected based on the *Discosoma sp*. red fluorescent protein (DsRed1) fluorescence and fixed in ice cold 90% methanol for two hours at -20 ºC. The roots were washed three times for 10 min in reaction buffer containing 0.1 M Na_2HPO_4-NaH_2PO_4 pH7 and incubated in reaction buffer supplemented with 1 mM $K_3Fe(CN)_6$, 1 mM $K_4Fe(CN)_6$, 5 mM NaEDTA, 0.1% Triton X-100 and 2 mM 5-bromo-4-chloro-3-indolyl-beta-D-glucuronide. Samples were gently vacuum-infiltrated for 30 min and then incubated at 37 °C, in the dark, for 24 hours. Samples were imaged with a DM6000 microscope (Leica).
To assess the localization in *M. truncatula* roots, *pCaM2:gCaM2* was subcloned into the pK7RWG2-KDEL:GFP (14). Subcellular localization of the CaM2:RFP in *M. truncatula* hairy roots was determined by confocal laser scanning microscopy using a Zeiss LSM780.

Co-Immunoprecipitation and Liquid chromatography-MS/MS assay. To identify CaM-CNGC15a interaction *in planta*, immunoprecipitation assay was performed using protein extracts from roots of R108/*p35S:CNGC15a:Myc:YFP*^{N}*:T35S* line and wild type R108 plants in presence of 5 mM $CaCl_2$ or 5 mM EDTA. 3 grams of roots were ground and solubilized in 10 mL of lysis buffer containing 10% glycerol, 50 mM TrisHCl 8.0, 150 mM NaCl, 1% Nonidet p40, 1 mM PMSF, 20 µM MG132 and 1x protease inhibitor mix (Roche cOmplete, EDTA free). The lysis buffer was supplemented with 5 mM $CaCl_2$ or 5 mM EDTA to assess CaM interaction in presence or absence of calcium, respectively. The samples were homogenized using a Potter-Elvehjem tissue homogenizer. After incubation on ice for 30 min, the homogenized samples were centrifuged three times at 3600 x g for 10 min under 4ºC. 50 µl of MYC-trap agarose beads (Chromotek), equilibrated in washing buffer (50 mM TrisHCl 8.0, 150 mM NaCl, 0.1 % Triton X-100 and 1x protease inhibitor mix (Roche cOmplete, EDTA free) supplemented with 5 mM $CaCl_2$ or 5 mM EDTA were added to each sample. After incubation for 1 hour at 4ºC with continuous shaking, the beads were centrifuged at 2000 x g for 2 min and washed three times with 1 mL of washing buffer. Finally, the bound proteins were eluted by incubating the beads for 10 min with 100 µL of SDS loading buffer (100mM TrisHCl 6.8, 4% SDS, 20% glycerol, 2% β-mercaptoethanol, 25mM EDTA, 0.04% bromophenol blue) at 95 ºC. The eluted fraction was centrifuged 2 min at 2700 x g, and the supernatant collected. To assess the presence of CNGC15a-MYC-nYFP, 20 µL of the fraction was separated on a 4-20% precast polyacrylamide gel (BIO-RAD: 4561094) and immunoblotted with an anti N-term GFP antibody (Sigma G-1544, dilution 1/1000) and anti-rabbit IgG-HRP (Santa Cruz Biotechnology, dilution 1/10000).

Two biological replicates were obtained and the CNGC15a:MYC coimmunoprecipitated proteins contained within gel slices were prepared as previously described (15). The aliquots were analyzed using nano LC-MS/MS on an Orbitrap Fusion™ Tribrid™ mass spectrometer coupled to an UltiMate® 3000 RSLCnano LC system (Thermo Fisher Scientific). The samples were loaded and trapped using a pre-column with 0.1% TFA at 20 µL min^{-1} for 3 min. The trap column was then switched in-line with the analytical column (nanoEase M/Z column, HSS C18 T3, 100 Å, 1.8 µm; Waters, Wilmslow, UK) for separation using the following long gradient of solvents A (water, 0.05% formic acid) and B (80% acetonitrile, 0.05% formic acid) at a flow rate of 0.3 µL min^{-1} : 0-4 min 3% B (trap only); 3-13 min linear increase B to 13%; 13-77 min increase B to 38%; 77-92 min increase to 55%; 92-97 min increase B to 55%; followed by a ramp to 99% B and re-equilibration to 3% B. Data were acquired with the following mass spectrometer settings in positive ion mode: MS1/OT [resolution 60K, profile mode, mass range *m/z* 300-1800, AGC $4e^5$, fill time 50 ms], MS2/IT [data analysis was performed using HCD and CID fragmentation with the following parameters: top20 in IT rapid, centroid mode, isolation window

1.6 Da, charge states 2-5, threshold $1.9e^4$, CE = 30, AGC target $1.9e^4$, max. inject time 35 ms, dynamic exclusion 1 count, 15 s exclusion, exclusion mass window ±5 ppm].
Peaklists were generated with MaxQuant 1.6.17.0 (16) in LFQ mode using the Medicago protein sequence database [Medicago truncatula, BioProject 10791, (57,585 entries)] and the MaxQuant contaminants database (245 entries). The quantitative LFQ results from MaxQuant with default parameters were used together with search results from an in-house Mascot Server 2.4.1 (Matrixscience, London, UK) on the same databases. A precursor tolerance of 6 ppm and a fragment tolerance of 0.6 Da was used. The enzyme was set to trypsin/P with a maximum of 2 allowed missed cleavages; oxidation (M), acetylation (protein N-term) was set as variable modifications; carbamido-methylation (C) as fixed modification. The Mascot search results were combined into Scaffold 4 (www.proteomesoftware.com) using identification probabilities of 99% for proteins and 95% for peptides.

Bimolecular fluorescence complementation of CNGC15a and CaM2 in *M. truncatula* roots. The transgenic *M. truncatula* R108/*p35S:CNGC15a:Myc:YFP^N:T35S* line was transformed with *A. rhizogene* AR1193 carrying the golden gate constructs *pCaM2:YFP^C:CaM2:TNOS-p35S:mCherry:T35S* or *pCaM2:YFP^C:TNOS- p35S:mCherry:T35S* (Sup. Fig. 16). Two-weeks old transformed roots were analyzed for yellow fluorescent protein (YFP) and mCherry fluorescence using the confocal laser scanning microscope Zeiss LSM780, (YFP: excitation 514nm, emission imaged between 520 and 580nm; mCherry: excitation 587nm, emission imaged between 600 and 620nm). Three biological replicates were analyzed for each construct.

Protein expression and purification. The coding sequence of *CaM2*, *CaM2^{R91A}* and the C-terminal sequence of *CNGC15a, CNGC15b* and *CNGC15c* were cloned into the expression vector pOPIN-M (17) by In-Fusion™ reaction (Clontech-Takara). All sequences were PCR-amplified from the yeast two hybrid pBD and pAD clones generated in this study using the primers listed in Dataset S2. The resulting constructs were transformed into *Escherichia coli* Rosetta™ (DE3) pLysS™ (18), and the expression of the N-terminal His6-MBP-3C tagged proteins were induced by 1 mM isopropylthio-β-D-1-galactopyranoside (IPTG) when the culture reached an OD_{600} of 0.5 at 30 °C. The IPTG-induced cultures were incubated at 16 °C for 14h before collecting the cells by centrifugation at 5663 x g for 10 min and resuspending in 100 mL (ratio: 1/80; volume of buffer/volume of cell culture) of 50 mM Tris-HCl (pH 7.5), 500 mM NaCl, 50 mM glycine, 5% (v/v) glycerol, 20 mM imidazole supplemented with EDTA-free protease inhibitor tablets (Roche cOmplete, EDTA free). Cells were sonicated and following centrifugation at 38,724 x g for 45 min at 4°C, the N-terminal His6-MBP-3C tagged proteins present in the lysate were purified via immobilized metal affinity chromatography using a 5 mL Ni^{2+}-NTA column (GE Healthcare), followed by gel filtration on a Superdex™ 200 16/60 column (GE Healthcare). For BLItz and ITC, the MBP tag was cleaved from His6-MBP-3C: CaM2 and His6-MBP-3C: CaM2^{R91A}, by 3C protease (1:100 ratio of protein:protease) treatment at 4 °C for 16 hours. The cleaved proteins were purified using tandem Ni^{2+}-NTA and MBP Trap HP columns (GE Healthcare), followed by gel filtration on a Superdex™ 200 16/60 for final purification and buffer exchange with 20 mM HEPES pH 7.5, 150 mM NaCl buffer. Recombinant protein purity was assessed by SDS-PAGE gel (Sup. Fig. 17).

Biolayer interferometry. Biolayer interferometry (BLItz) was performed to monitor interactions of CaM2 or CaM2^{R91A} with the C-terminal domain of CNGC15s using BLItz instrument (FortéBio, Menlo Park, CA) and Ni-NTA Biosensors (FortéBio) at room temperature. The Ni-NTA biosensor was hydrated in reaction buffer (20 mM HEPES pH 7.5, 150 mM NaCl) for 10 min before each run. For each run, a baseline was established in the reaction buffer (20 mM HEPES pH 7.5, 150 mM NaCl) for 30 s, then 50 µM of His6-MBP:CNGC15a, His6-MBP:CNGC15b or His6-MBP:CNGC15c was loaded onto the Ni-NTA biosensor tip for 2 min. A baseline was re-established in the reaction buffer (20 mM HEPES pH 7.5, 150 mM NaCl) for 45 s to wash the unbound CNGC15s, followed by a control run in the presence of $CaCl_2$ (at concentration indicated) with an association step of 20s followed by a dissociation step of 20s. The CaM2 binding to CNGC15s was tested using purified CaM2 at the concentration indicated, in absence or presence of $CaCl_2$, with an association step of 20s followed by a dissociation step of 20s.

To calculate the Kinetics parameters (K_d, k_{on}, k_{off}, R^2), the corresponding control runs were subtracted, and the final values were determined using a 1:1 Binding Model using BLItz Pro software v1.2 (ForteBio). The replicates were performed with proteins from various number of independent expressions and purifications as indicated.

Isothermal Titration Calorimetry. Isothermal Titration Calorimetry (ITC) was carried out using a MicroCal PEAQ-ITC (Malvern Panalytical). To test the interaction of CaM2 and CaM2^{R91A} with calcium, the purified proteins were loaded at the final concentration of 100 µM in reaction buffer (20 mM HEPES pH 7.5, 150 mM NaCl). CaM2 and CaM2^{R91A} were titrated in the reaction buffer with 5 mM $CaCl_2$. Titration was scheduled with 18 consecutive injections of 2 µL of reaction buffer with 5 mM $CaCl_2$, with a 150 s interval between injections. To test the interaction of CaM2 and CaM2^{R91A} in presence of 5 mM $CaCl_2$ with the IQ domains of CNGC15b and CNGC15c, the custom peptides AACFIQVAWRRTIQEKKG for CNGC15b, and AACFIQAAWRRHKKRKEA for CNGC15c, were synthesized (Merck). For each assay, 10 µM of peptide diluted in the reaction buffer (20 mM HEPES pH 7.5, 150 mM NaCl, 5 mM $CaCl_2$) was titrated through 18 consecutive injections of 2 µL of 150 µM of CaM2 or CaM2^{R91A} in 20 mM HEPES pH 7.5, 150 mM NaCl and 5 mM $CaCl_2$. Data acquisition and analysis were performed using MicroCal PEAQ-ITC Software (Malvern Panalytical). The replicates were performed with CaMs purified from two independent proteins expressions and purifications.

Structural homology modelling and prediction of calmodulin mutant variant stability and flexibility. Homology models of CaM2 wild type and mutant were generated by Swiss Model using PDB 5a2h AtCaM7 as a template. The molecule stability and flexibility of the CaM2 mutants was predicted by submitting the model to the DynaMut server (19) (biosig.unimelb.edu.au/dynamut/). Figures were produced in CCP4mg (20).

Molecular cloning to test the effect of *CaM2* and *CaM2^{R91A}* in *M. truncatula* roots. The *NLS:YC3.6-Cherry*, *NLS:YC3.6-CaM2-mCherry* and *NLS:YC3.6-CaM2^{R91A}-mCherry* constructs were generated via GoldenGate cloning according to (21). Assembled level-1 modules were cloned in a level-2 binary vector backbone as presented in Sup. Fig. 10. Level 0 modules were synthetized by Life technologies™ (ThermoFisher Scientific) with the exception of *CaM2* and *CaM2^{R91A}* which were PCR amplified using the primers P49/P50 (Dataset S2). *RNAiCaMs* was PCR amplified from R108 gDNA and cloned into pK7GWIWG2D(II)R by BP/LR gateway cloning (Invitrogen) using primers P51/P52 (Dataset S2).

Calcium oscillation analyses. Calcium oscillation measurements were performed using a Nikon ECLIPSE FN1 equipped with an emission image splitter (OptoSplit II, Cairn Research) and an electron multiplying cooled charge coupled (Rolera™ Thunder EMCDD) camera (QImaging). ECFP was excited using light emitting diode (OptoLED, Cairn) at 436±20 nm and emitted fluorescence detected at 535±30 nm (cpVenus) and 480±40 nm (ECFP). Images were collected in 3s intervals for 1h30 using MetaFluor software. *M. truncatula* R108 roots expressing *NLS:YC3.6*, *NLS:YC3.6-CaM2* or *NLS:YC3.6-CaM2^{R91A},* and *M. truncatula* R108::YC3.6 roots expressing the empty pK7GWIWG2D(II)R or the pK7GWIWG2D(II)R::*RNAiCaMs* were generated as described above. 2 weeks old transformed plants were placed in a chamber made on a 48x64 mm coverglasses (Solmedia) using high-vacuum grease (Dow Corning GMBH). The chamber was filled with 2 mL of Buffered Nodulation Medium (BNM) (22). Only the root was covered with a coverslip to leave space for Nod factor application at a final concentration of 10^{-8} M. Nod factor was produced as previously described (23). Calcium imaging was performed on 2 cm long roots and on the root hair of the induction zone. To obtain a high quality of calcium recording, a maximum of two nuclei were imaged per transformed plants. Calcium oscillation traces were analyzed using GNU Octave v6.1.0 with the nan v3.5.0 and io v2.6.3 packages. The script developed to analyze the frequency, amplitude, rise time and fall time of the calcium spikes is included in Supplemental Dataset 3.

Arbuscular mycorrhiza, nodulation and infection assays. *M. truncatula* R108 plants which regenerated roots expressing *NLS:YC3.6*, *NLS:YC3.6-CaM2* or *NLS:YC3.6-CaM2^{R91A}* were selected by fluorescence microscopy and grown in controlled environment rooms at 22 °C (80% humidity, 16 h photoperiod, 300 μmol m^{-2} s^{-1} light intensity). To monitor AM colonization, plants were grown in Terragreen/Sand (Oil-Dri Company, Wisbech, UK) and inoculated with *R. irregularis* (Endorize; Agrauxine, France) to the ratio 5:5:1 (Terragreen/Sand/Spores). Colonization was monitored over time in the wild type R108:: *NLS:YC3.6* control, and colonization of the assays was analyzed when the wild type reach 10%, 30% and 50% of arbuscules development, named early, mid and late stages of colonization, respectively. Early, mid and late stages of colonization correspond to 20-, 36-, and 50-days post inoculation, respectively. The fungal structures were stained in acidic ink as follows; roots were cleared in 10% KOH for 15 min at 95°C, washed 3 times in water and subsequently stained in acidic ink (5% ink, 5% acetic acid) for 4 min at 95°C. The AM root length colonization was quantified using the grid intersect method (24). For nodulation assays, plants were grown in Terragreen/Sand (Oil-Dri Company, Wisbech, UK) to a ratio (1:1) for 7 days and then inoculated with *S. meliloti* 2011 (OD_{600}=0.01). Nodulation was scored after inoculation as indicated. Infection assays were performed as previously described (25).

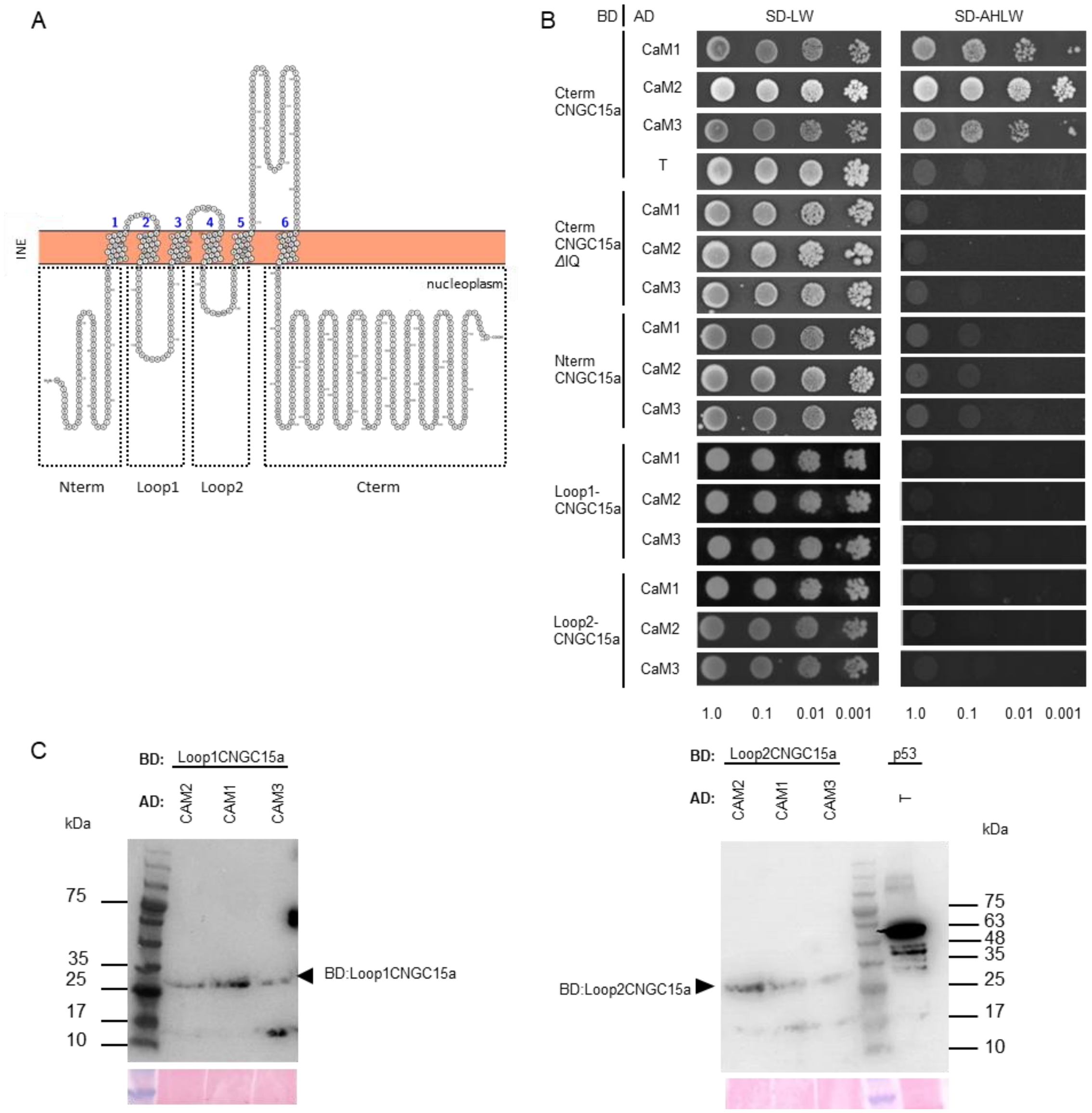

Fig. S1. CaMs interact specifically with the Cterminal domain of CNGC15a.

(A) Predicted transmembrane (TM) domain topology of CNGC15a using Protter (https://wlab.ethz.ch/protter/). Blue numbers indicate the TM. Dashed rectangle indicates the domains within the nucleoplasm and used to assess CaMs binding via pairwise yeast two hybrid. INE: Inner nuclear envelope. TM prediction tools cannot predict the topology of ion channel pore in the membrane; hence, the topology of CNGC15a pore between TM5 and TM6 is not represented. (B) GAl4-based yeast two-hybrid assays to assess pairwise interactions between the nucleoplasmic domain of CNGC15a including the C-terminal (Cterm) domain, Cterm with the deletion of the calmodulin binding domain 613-AACFIQAAWFRYKRMKE-629 (CNGC15$^{\Delta IQ}$), N-terminal (Nterm) domain, the loop 1 and the loop2 used as bait (BD), and CaM1, CaM2 and CaM3 as prey (AD). The SV40 large T-antigen (T) is used as negative control. Dilution series of co-transformants were plated on synthetic dropout (SD) medium lacking leucin (L) and tryptophan (W) and SD lacking adenine (A), histidine (H), L and W. (C) Western blot analyses to assess the expression of BD:Loop1CNGC15a (25.8 kDa) and BD:Loop2CNGC15a (26.2 kDa). (Additional expression analyses in Sup. Fig. 10). Proteins are detected with anti-GAL4:BD antibodies. The detection of BD:53 (69.8 kDa) from the protein extract of yeast co-expressing BD:53 and AD:T was used as a positive control (Supplemental figure 10). Arrowhead indicates the targeted proteins. Bottom panel: Ponceau staining of the membrane after western blotting.

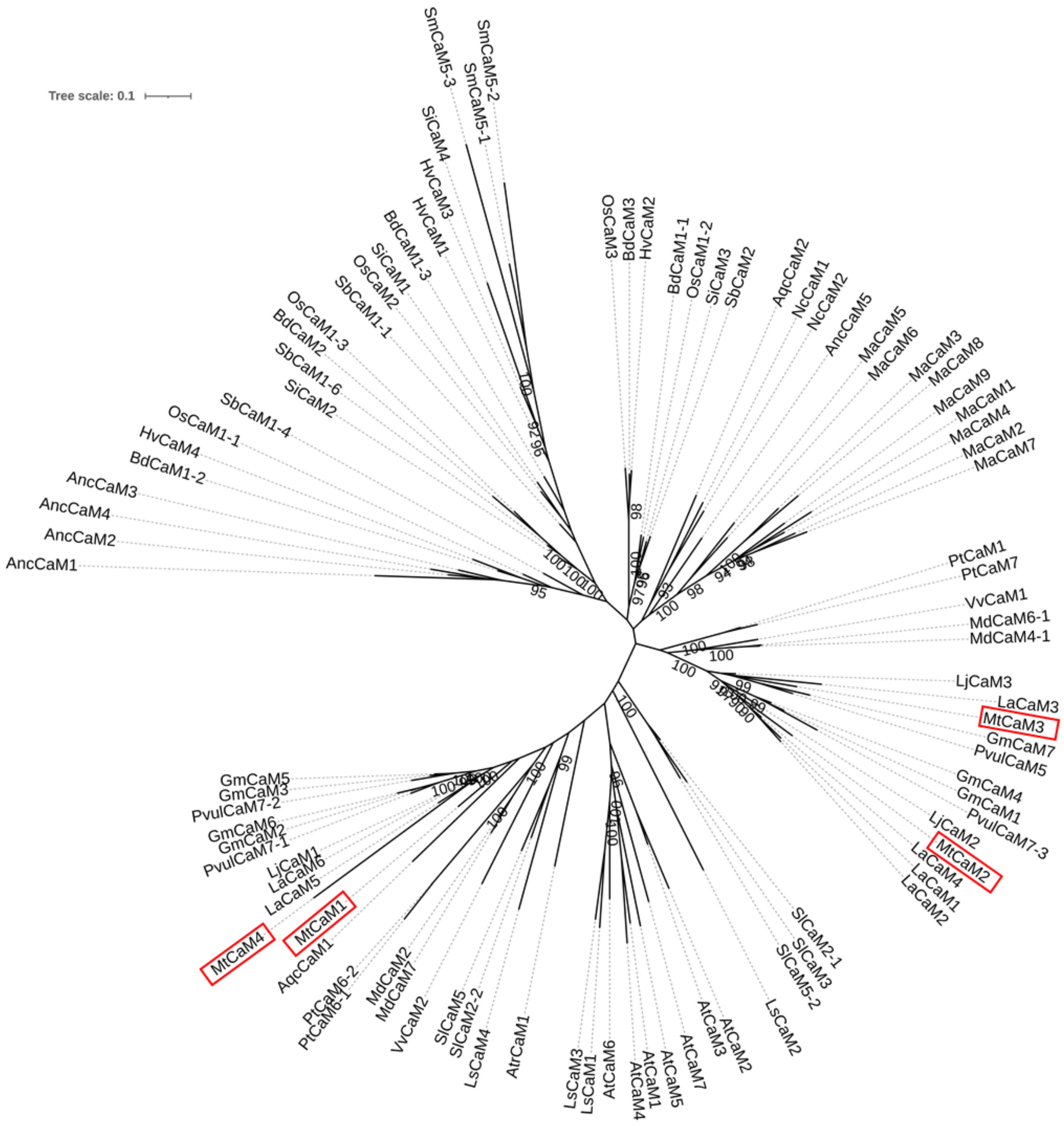

Fig. S2. Phylogenetic analyses of the *Medicago truncatula CaMs.*

Unrooted maximum likelihood tree using DNA coding sequences under the TPM2u+R5 codon model. The sequences used are available in Dataset S1. Ultrafast bootstrap support values over 90 are labelled on the branches. Species abbreviation - Anc: *Ananas comosus*; At: *Arabidopsis thaliana*; Atr: *Amborella trichopoda*; Aqc: *Aquilegia coerulea*; Bd: *Brachypodium distachyon*; Gm: *Glycine max*; Hv: *Hordeum vulgare*; La: *Lupinus albus*; Lj: *Lotus japonicus*; Ls: *Lactuca sativa*; Ma: *Musa acuminata*; Md: *Malus domestica*; Mt: *Medicago truncatula*; Nc: *Nymphaea colorata*; Os: *Oryza sativa*; Pt: *Populus trichocarpa*; Pv: *Phaseolus vulgaris*; Sb: *Sorghum bicolor*; Si: *Setaria italica*; Sl: *Solanum lycopersicum*; Sm: *Selaginella moellendorffii*; Sl: *Solanum lycopersicum*; Vv: *Vitis vinifera*. Red rectangle indicates *M. truncatula CaMs.*

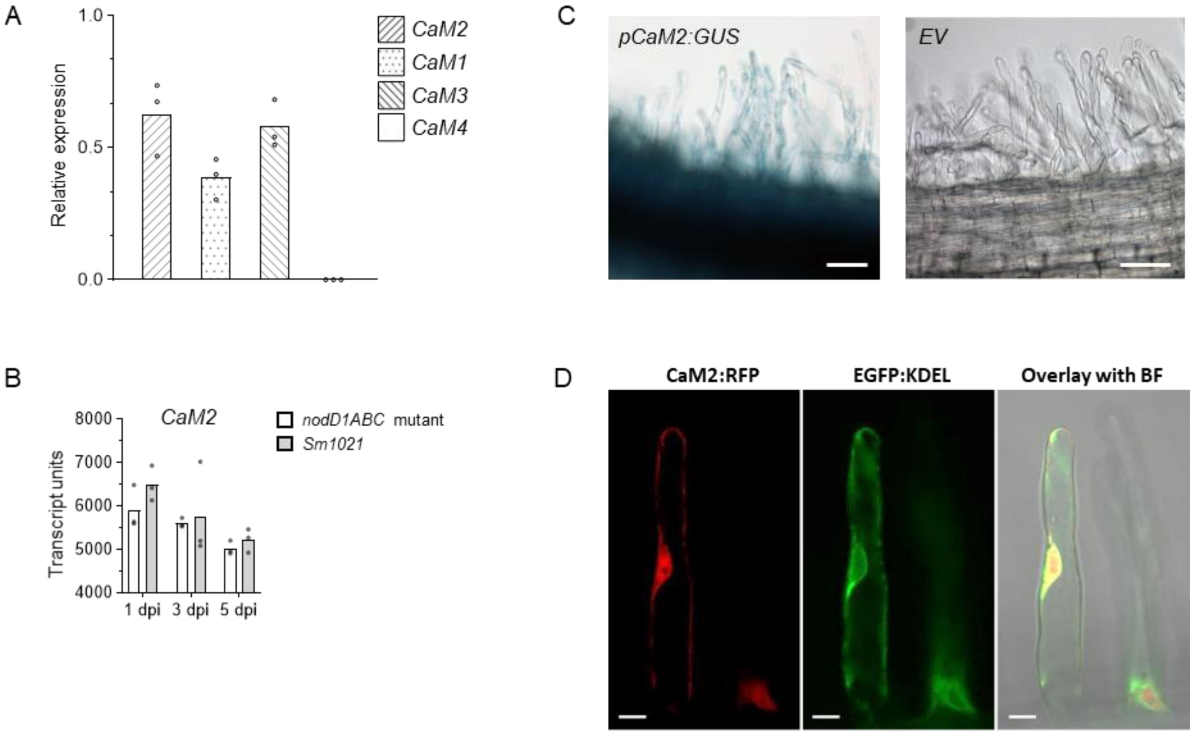

Fig. S3. Expression of *CaM2* and localization of CaM2 in *M. truncatula* roots.

(A) Quantitative expression analysis by RT-qPCR of the transcript level of the *CaM1, CaM2, CaM3 and CaM4* in non-inoculated roots at 20 days post germination. Expression is normalized to the *UBC9* (*TC106312*) (n=3). (B) Expression analyses of *CaM2* in *M. truncatula* root hair based on available microarray data (*M. truncatula* gene expression atlas: MtGEA; http://mtgea.noble.org/v3/). Normalized average expression of the probe *Mtr.48177.1.S1_at* specific to *MtCaM2* was assessed after 1, 3 and 5 days post inoculation (dpi) with the wild type *Sinorhizobium meliloti 1021* and the deletion mutant *Sm1021 nodD1ABC* which cannot produce Nod factor (n=3). (C) β-glucuronidase (*GUS*) activity in 2 weeks old *A. rhizogenes* transformed roots expressing *pCaM2* fused to *GUS in non-inoculated roots.* Scale bar = 60 µm. (D) Localization of CaM2 fused to the Red Fluorescence Protein (RFP) in the nucleus and the cytoplasm. *CAM2:RFP* is expressed under the promoter *CAM2. CaM2* is co-expressed with the endoplasmic reticulum marker *EGFP:KDEL*. Left panel: red channel, middle panel: green channel, right panel: overlay with bright field (BF). Scale bar = 10 µm.

A

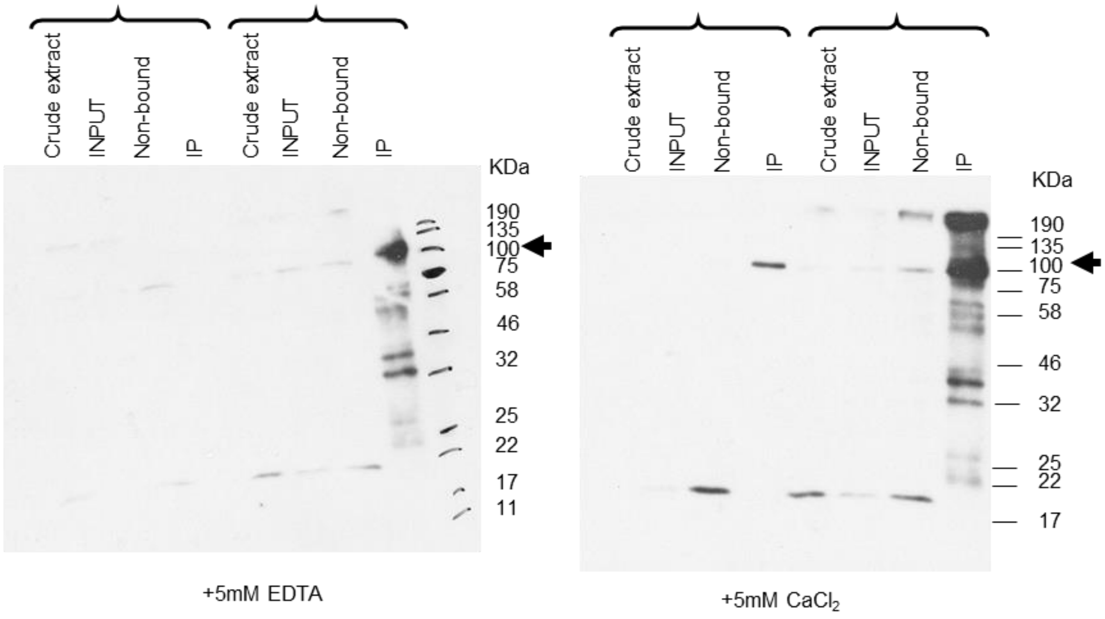

B

```
CaM4_Medtr7g087610      MADQLTDDQISEFKEAFSLFDKDGDGSITTKELGTVMRSLGQNPTEAELQDMINEVDADG   60
CaM3_Medtr0168s0120     MADQLTDDQIAEFKEAFSLFDKDGDGCITTKELGTVMRSLGQNPTEAELQDMINEVDADG   60
CaM2_Medtr5g088320      MADQLTDEQISEFKEAFSLFDKDGDGCITTKELGTVMRSLGQNPTEAELQDMINEVDADG   60
CaM1_Medtr6g025320      MADQLTDDQISEFKEAFSLFDKDGDGCITTKELGTVMRSLGQNPTEAELQDMINEVDADG   60
                        *******:**:***************.*********************************

CaM4_Medtr7g087610      NGTIDFPEFLNLMARKMKDTDSEEELKEAFRVFDKDQNGFISAAELRHVMTNLGEKLTDE   120
CaM3_Medtr0168s0120     NGTIDFPEFLNLMARKMKDTDSEEELKEAFRVFDKDQNGFISAAELRHVMTNLGEKLTDE   120
CaM2_Medtr5g088320      NGTIDFPEFLNLMARKMKDTDSEEELKEAFRVFDKDQNGFISAAELRHVMTNLGEKLTDE   120
CaM1_Medtr6g025320      NGTIDFPEFLNLMARKMKDTDSEEELKEAFRVFDKDQNGFISAAELRHVMTNLGEKLTDE   120
                        ************************************************************

CaM4_Medtr7g087610      EVDEMIREADVDGDGQINYDEFVKVMMAK*          149
CaM3_Medtr0168s0120     EVDEMIREADVDGDGQINYEEFVKVMMAK-          149
CaM2_Medtr5g088320      EVDEMIREADVDGDGQINYEEFVKVMMAK*          149
CaM1_Medtr6g025320      EVDEMIREADVDGDGQINYEEFVKVMMAK*          149
                        *******************:*********
```

Fig. S4. Targeted immunoprecipitation coupled with mass spectrometry analyses of CaMs using CNGC15a:Myc:nYFP as bait.

(A) Immunoprecipitation and blot analyses of the protein fractions from roots of *Medicago truncatula* wild-type R108 and R108 transgenic line expressing *CNGC15a:Myc:nYFP* treated with 5 mM EDTA or 5 mM $CaCl_2$. CNGC15a:Myc:nYFP (103 kDa) and its ligand proteins were immunoprecipitated using MYC-trap agarose beads and detected using Nterm-GFP antibody (Sigma G-1544). Black arrows indicate CNGC15a:Myc:nYFP. (B) Alignment of the CaMs amino acid sequences. Mass spectrometry analyses revealed peptides of CaM2 (17 kDa) (shaded in yellow).

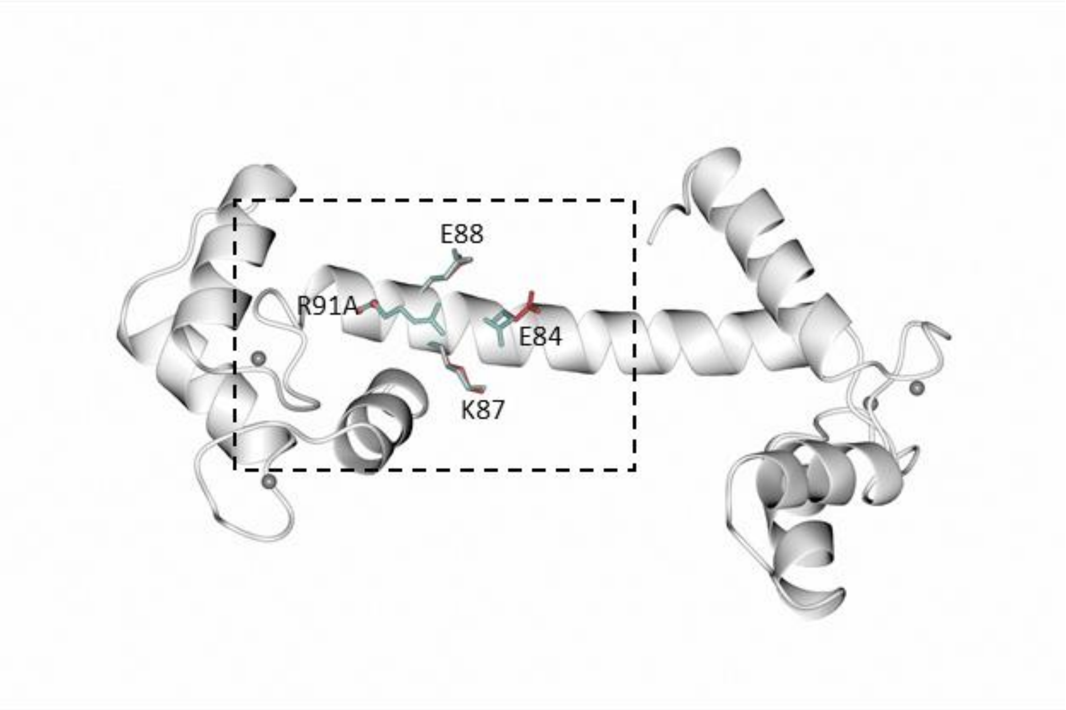

Fig. S5. Structure homology models overlay of CaM2 and CaM2^{R91A}

The position of the residues that form contacts with R91 are shown in blue. The position of the residue in R91A is indicated in red. The dashed rectangle indicates the position of the region of CaM2 and CaM2^{R91A} zoomed and presented in Figure 2A.

A

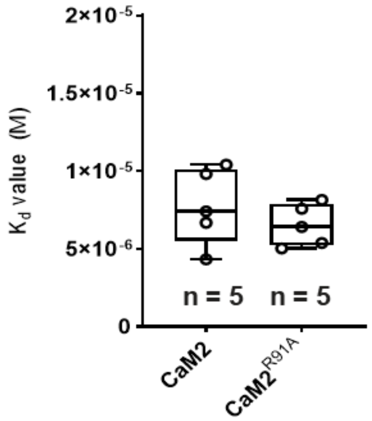

B

Construct	K_d	n	ΔH	-TΔS	ΔG
	μM		kJ/mol	kJ/mol	kJ/mol
CaM2	7.72 ± 2.47	3.39 ± 4.80e-1	$5.1 \pm 8.22e^{-1}$	-34.44 ± 1.7	$-29.32 \pm 8.53e^{-1}$
$CaM2_{R91A}$	6.5 ± 1.35	3.9 ± 5.98e-1	$3.92 \pm 4.17e^{-1}$	$-33.58 \pm 9.01e^{-1}$	$-29.7 \pm 5.1e^{-1}$

Fig. S6. Thermodynamic parameters of the isothermal titration calorimetry measurements of the interaction of CaM2 and $CaM2^{R91A}$ with calcium.

(A) K_d values obtained from five independent ITC measurements performed with proteins from two independent purifications. Student's t-test. No statistical differences were observed for the K_d of calcium for CaM2 and the K_d of calcium for $CaM2^{R91A}$. (B) Detailed thermodynamic parameters of calcium binding to CaM2 and $CaM2^{R91A}$**.** Values correspond to the mean of five independent experiments. n: stoichiometry, ΔH: enthalpy, ΔS: entropy, ΔG: free energy value expressed as ΔG= ΔH –TΔS.

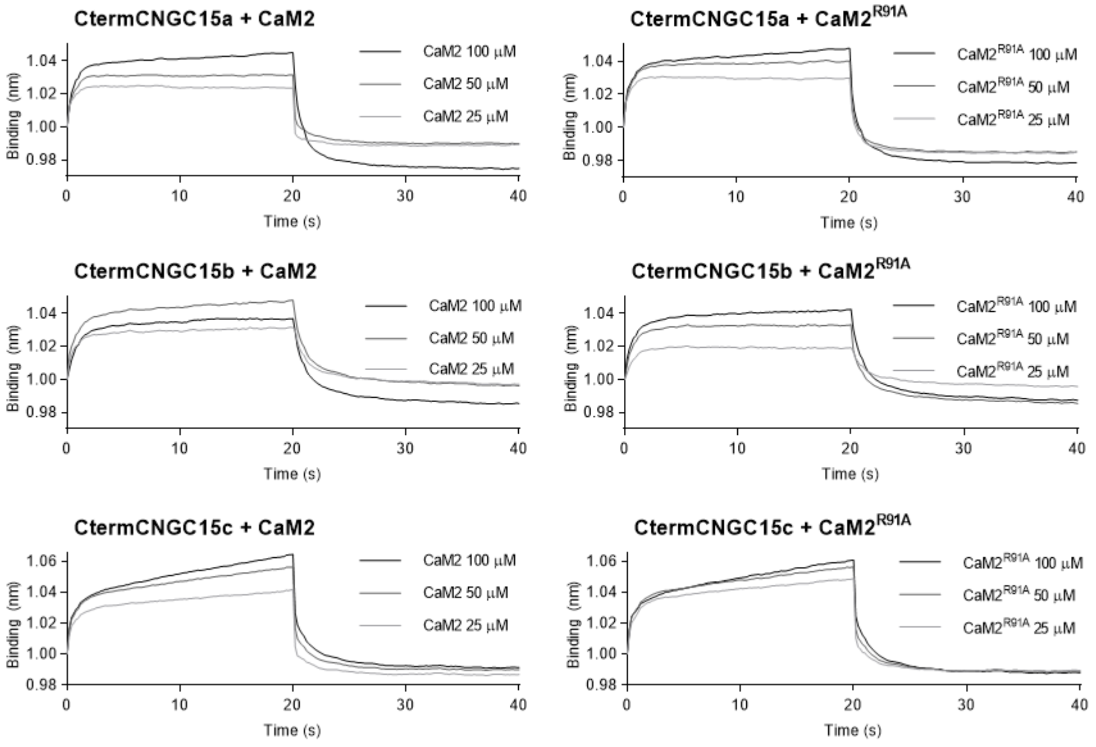

Fig. S7. Biolayer interferometry graphs of CaM2 and CaM2^{R91A} binding to CtermCNGC15s in presence of calcium.

Biolayer interferometry experiments of CaM2 and CaM2^{R91A} binding to CtermCNGC15a, CtermCNGC15b and CtermCNGC15c in presence of 20 mM $CaCl_2$. A concentration range of CaM (25, 50 and 100 µM) was used for each experiment. The curves represent the average of three replicates normalized to control run, and performed using one protein purification. Graphs show the association (0-20 s) and disassociation (20-40 s) steps of the interaction.

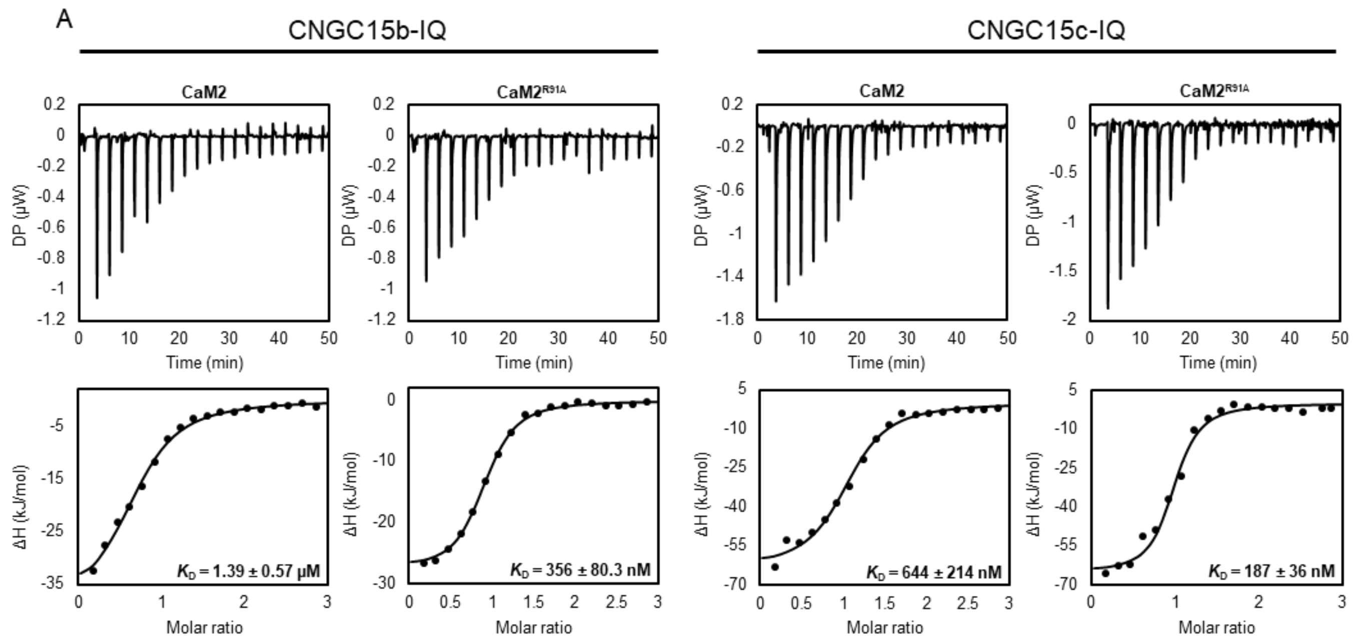

B

Construct	K_d	n	ΔH	-TΔS	ΔG
	µM		kJ/mol	kJ/mol	kJ/mol
CaM2	1.39 ± 0.57	0.69 ± 5.91e-2	-39.93 ± 5.36	6.33 ± 6.29	-33.6 ± 9.64e^{-1}
CaM2$_{R91A}$	0.36 ± 0.08	0.88 ± 1.29e-1	-27.6 ± 2.17	-9.26 ± 2.67	-36.9 ± 5.29e^{-1}

C

Construct	K_d	n	ΔH	-TΔS	ΔG
	µM		kJ/mol	kJ/mol	kJ/mol
CaM2	0.64 ± 0.21	1.04 ± 2.31e-2	-63.7 ± 8.65	28.2 ± 9.4	-35.47 ± 7.51e^{-1}
CaM2$_{R91A}$	0.19 ± 0.04	0.91 ± 6.08e-2	-60.6 ± 2.26	22.15 ± 2.76	-38.45 ± 4.95e^{-1}

Fig. S8. Isothermal titration calorimetry measurements of CaM2 and CaM2^{R91A} binding to the IQ domains of CNGC15b and CNGC15c in presence of calcium.

(A) Isothermal titration calorimetry of CaM2 (150 µM) and CaM2^{R91A} (150 µM) with 10 µM of the peptides corresponding to the IQ domains of CNGC15b and CNGC15c in presence of 5 mM $CaCl_2$. Top panel shows the average of the representative thermograms after baseline correction and noise removal obtained for automatic injections of CaM2 or CaM2^{R91A} over time. Bottom panels show the average of the integrated curve of the experimental points (black circles). (B-C) Thermodynamic parameters of the CaM2 and CaM2^{R91A} binding to the IQ motifs of CNGC15b (B) and CNGC15c (C)**.** Values correspond to the mean of three independent experiments using two independent protein purifications.

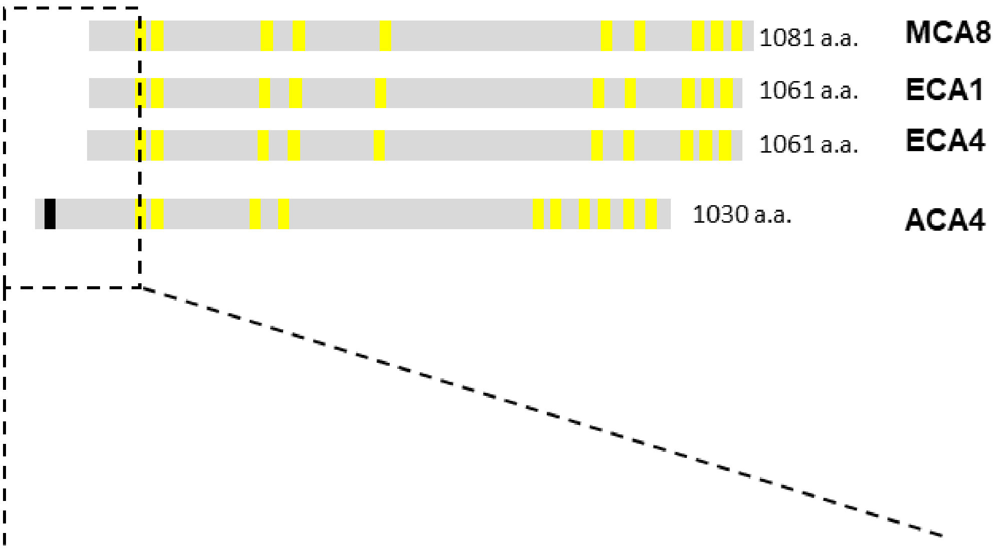


```
                                    CAMBD
AtACA4      MSNLLRDFEVEAKNPSLEARQRWRSSVSIVKNRTRRFRNIRDLDKLADYENKKHQIQEKI   60
MCA8        ------------------------------------------------------------   0
AtECA4      ------------------------------------------------------------   0
AtECA1      ------------------------------------------------------------   0

AtACA4      RVAFFVQKAALHFIDAAARPEYKLTDEVKKAGFSIEADELASMVRKNDTKSLAQKGGVEE   120
MCA8        ---------------------------MGKGGENYGRKENTSSDNSDGEIFKAWSKDVRE   33
AtECA4      ---------------------------MGKGGEDCGNKETNSSELVKSDTFPAWGKDVSE   33
AtECA1      ---------------------------MGKGSEDLVKKESLNSTPVNSDTFPAWAKDVAE   33
                                       : *.. .   .*  .    ..    *   .* *
                                                                TM
AtACA4      LAKKVSVSLSEGIRSSEVPIREKIFGENRYTEKPARSFLMFVWEALHDITLIILMVCAVV   180
MCA8        CEEHFKVSVKTGLSHDEVENRRKIYGFNELEKHDGQSIWKLVLEQFNDTLVRILLAAAII   93
AtECA4      CEEKFGVSREKGLSTDEVLKRHQIYGLNELEKPEGTSIFKLILEQFNDTLVRILLAAAVI   93
AtECA1      CEEHFVVSREKGLSSDEVLKRHQIYGLNELEKPEGTSIFKLILEQFNDTLVRILLAAAVI   93
              ::. ** . *:  .**  *.:*:* *.  :  . *:  :: * ::*  : **:..*::
```

Fig. S9. The calcium ATPase IIA, MCA8, and its closest orthologs, AtECA1 and AtECA4.

Upper panel: Representation of the protein sequence of the *Medicago truncatula* MCA8 and its *Arabidopsis thaliana* (At) homolog ECA1 and ECA4. MCA8 belong to the calcium ATPase type IIA which is not regulated by calmodulin. MCA8 shares 78.2% and 78% identity with ECA1 and ECA4, respectively (LALIGN). The calcium ATPase type IIB, ACA4, regulated by calmodulin is represented. Bottom panel: alignment of the N terminus of MCA8 protein sequence with AtECA1, AtECA4 and AtACA4. The calmodulin binding domain (CAMBD) and the transmembrane domain (TM) are highlighted in black and yellow, respectively.

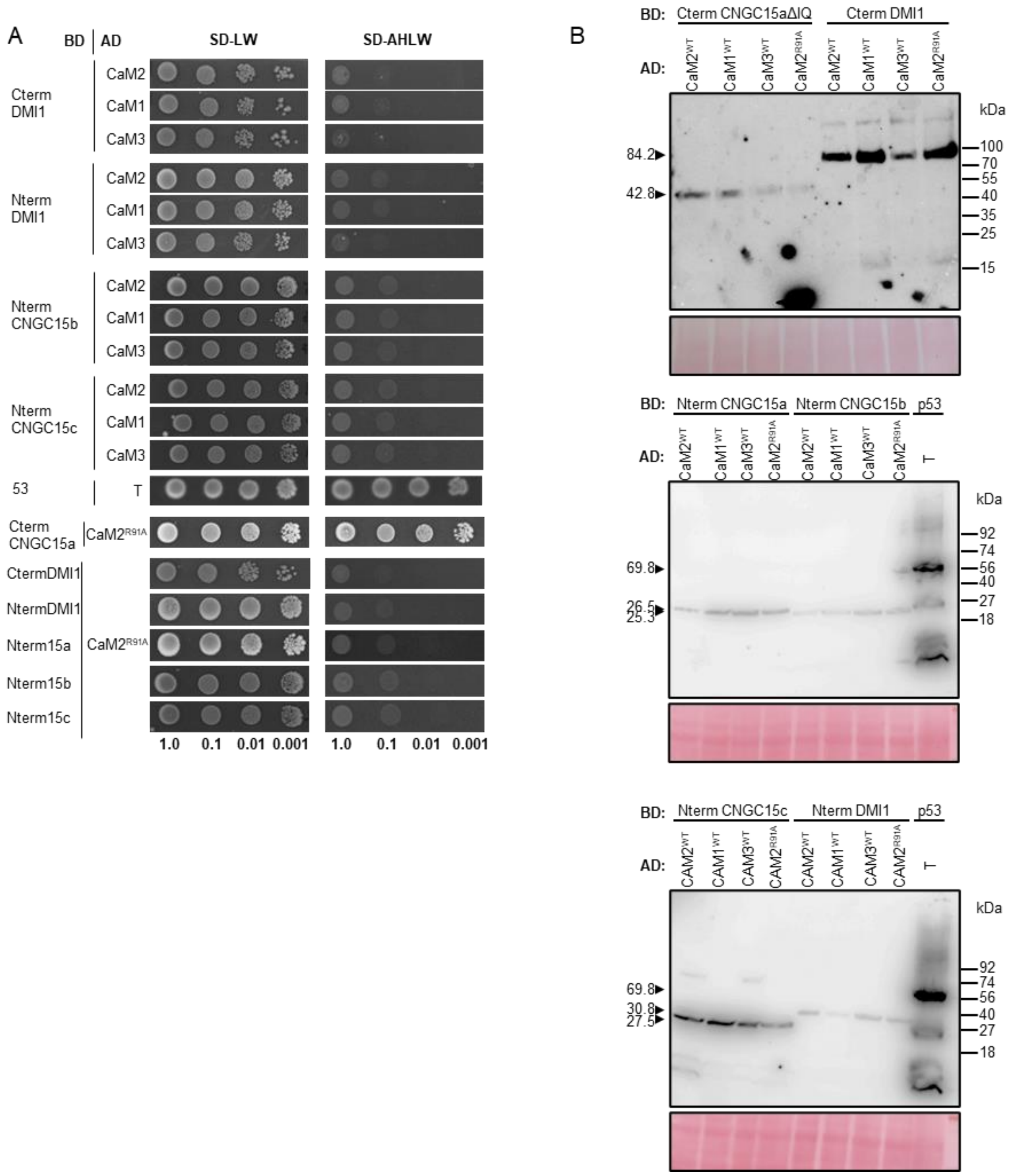

Fig. S10. CaMs and CaM2^{R91A} do not interact with DMI1 and NtermCNGC15s in yeast two hybrid assays.

(A) GAl4-based yeast two-hybrid (Y2H) assays to assess pairwise interactions between the C-terminal (Cterm) DMI1, Nterminal (Nterm) DMI1, NtermCNGC15b, NtermCNGC15c fused to the GAL4 Binding domain (BD) with CaM1, CaM2, CaM3 and CaM2^{R91A} fused to the GAL4 activator domain (AD). Y2H assays is also performed with BD:NtermCNGC15a and AD:CaM2^{R91A}. Co-transformant with BD:CtermCNGC15a and AD:CaM2^{R91A}, and BD:53 and AD:T were used as a positive control. Dilution series of the co-transformants were plated on synthetically dropout (SD) medium lacking leucin (L) and tryptophan (W), and on SD medium lacking adenine (A), histidine (H), and LW. Representative pictures of growth after 5 days incubation at 30ºC. Three replicates were performed. (B) Western blot analyses to assess the expression of BD:CtermCNGC15aΔIQ (42.8 kDa), BD:CtermDMI1 (84.2 kDa), BD:NtermCNGC15a (26.5 kDa), BD:NtermCNGC15b (25.3 kDa), BD:NtermCNGC15c (27.5 kDa) and BD:NtermDMI1 (30.8 kDa). Proteins are detected with anti-GAL4:BD antibodies. The expected size of the fusion proteins and the protein ladder are indicated on the left and right of each western blot, respectively. Bottom panel shows the proteins loading verified by Ponceau staining of the membrane after western blotting.

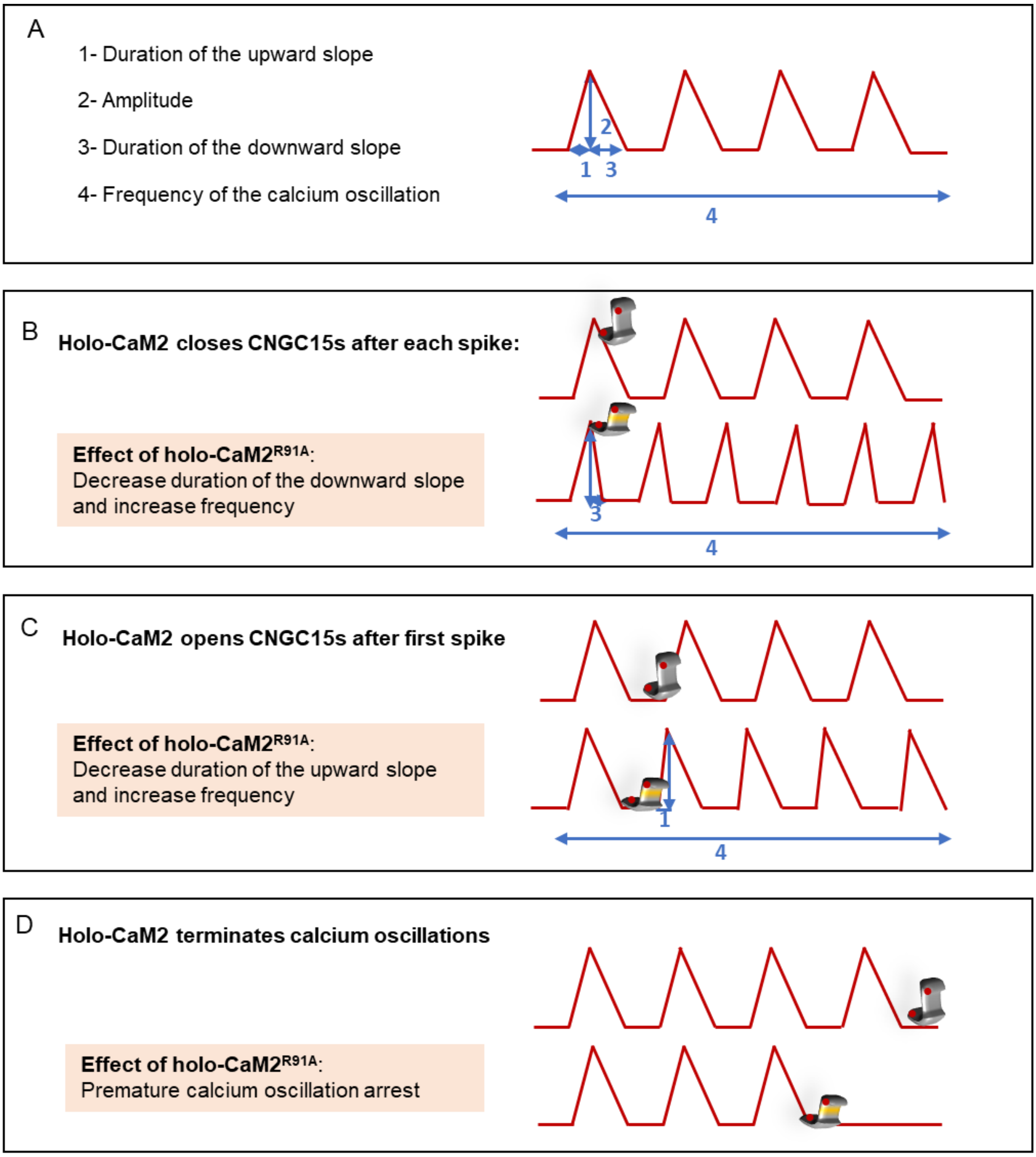

Fig. S11. Using engineered CaM2^{R91A} to unravel the role of CaM2 in modulating the nuclear calcium oscillation.

(A) Four parameters characterise the calcium oscillation pattern; the duration of the upward slope of each spike, the amplitude of each spike, the duration of the downward slope of each spike, and the frequency of the calcium oscillation. (B-D) Overexpressing CaM2^{R91A} which has an increase affinity for CNGC15s is used to unravel the function of CaM2. We hypothesise that (B) if holo-CaM2 closes CNGC15s, holo-CaM2^{R91A} will decrease the duration and the spike's downward slope and increase frequency of the oscillation, (C) if holo-CaM2 opens CNGC15s, holo-CaM2^{R91A} will decrease the duration and the spike's upward slope and increase frequency of the oscillation, and (D) if holo-CaM2 is involved in terminating the calcium oscillation, premature arrest of the calcium oscillation will be observed.

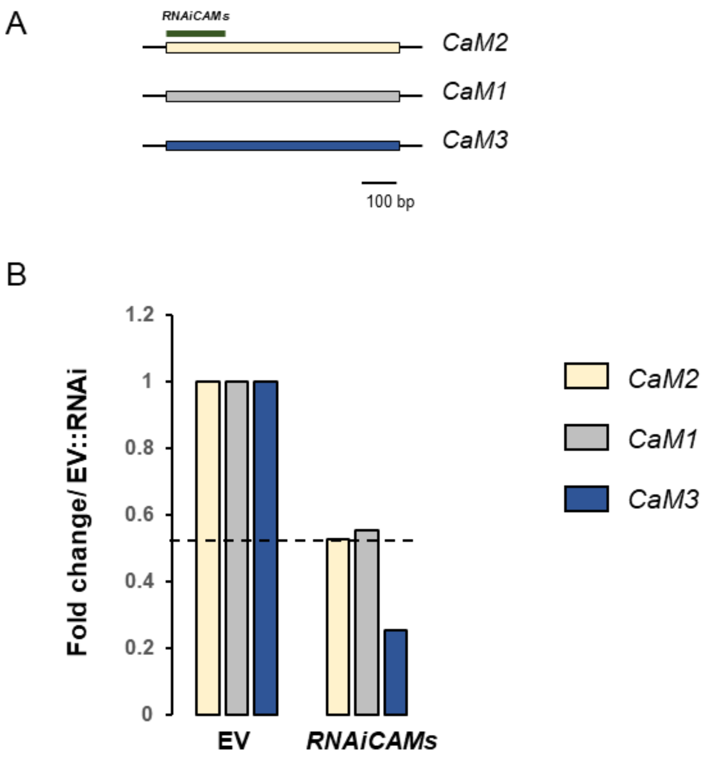

Fig. S12. Knockdown of *CaM2* via RNA interference

(A) Representation of the coding sequence of *CAM2* which shares 84% and 88.2% identity with *CaM1* and *CaM3,* respectively, and the position of RNAi construct. (B) Silencing level of *CaM2*, *CaM1* and *CaM3* in roots transformed via *Agrobacterium rhizogenes* mediated transformation with empty vector (*EV*) or *RNAiCaMs*. Expression level assessed via qRT-PCR is presented as the ratio relative to the expression in the empty vector control. The primers used in this study are included in Dataset S2.

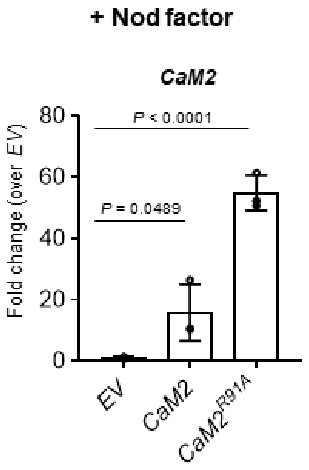

Fig. S13. Expression analyses of *CaM2* by qRT-PCR in hairy roots over-expressing the indicated genetic construct.

Hairy roots were harvested 6 hours after treatment with 10^{-8} M of Nod factor. Expression was normalised to *UBC9* (TC106312). *CaM2* expression is relative to *EV*. Bars, error bars and circles represent mean, standard deviation and individual values, (n=3). P-values (*P*) are shown (Dunnett's test, comparison to *EV*).

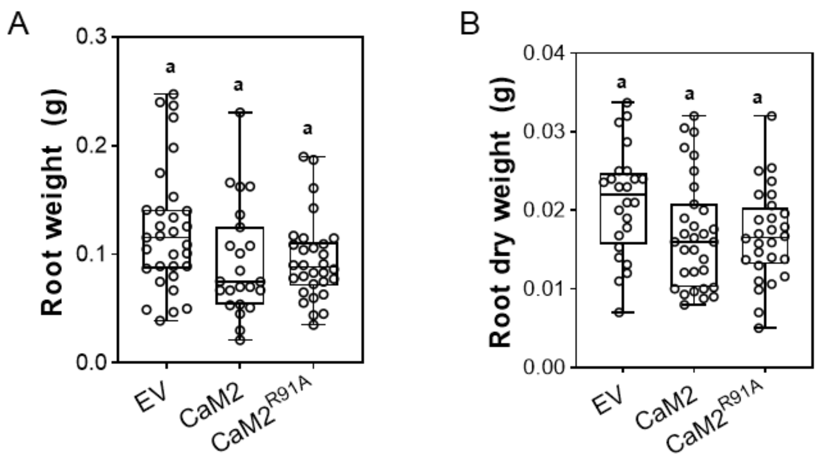

Fig. S14. Root weight of *M. truncatula* roots overexpressing CaM2^{R91A}.

Quantification of the root weight or root dry weight of *M. truncatula* roots overexpressing *CaM2*, *CaM2^{R91A}* or the empty vector (*EV*) after 14 days post inoculation (dpi) (A) and 28 dpi (B) with *Sinorhizobium meliloti* 2011. The samples were assessed for nodulation (Fig. 4B, D). Results represent the average of 3 biological replicates. (One-way ANOVA; posthoc Bonferroni); (A): n (*EV*) = 31, n (*CaM2*) = 23, n (*CaM2^{R91A}*) = 30; (B): n (*EV*) = 24, n (*CaM2*) = 31, n (*CaM2^{R91A}*) = 28.

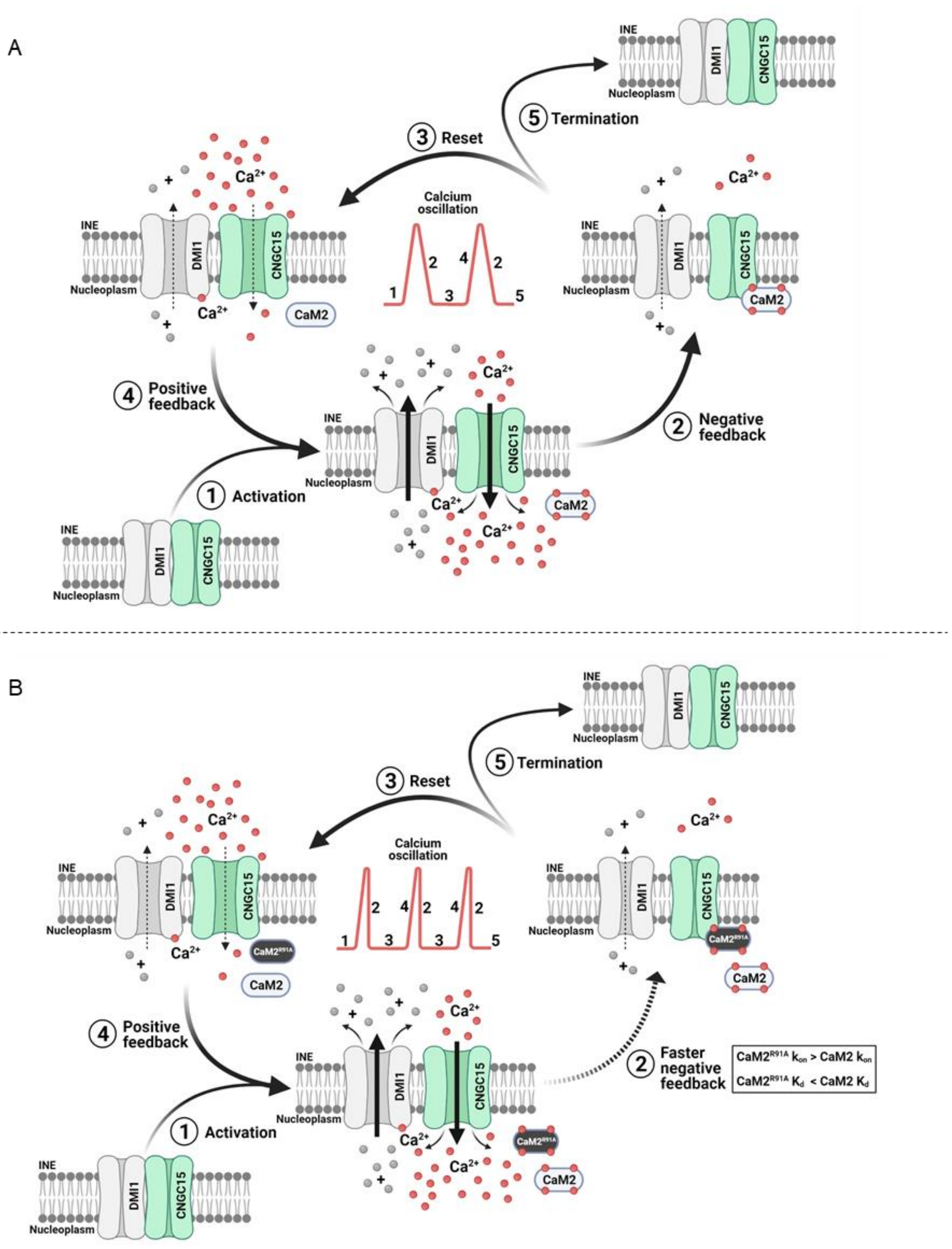

Fig. S15. Model of the calcium oscillatory mechanism.

(A) Upon a yet unknown activation mechanism (1) of either DMI1 or CNGC15, both ion channels undergo a structural change, putatively unlocking CNGC15 from DMI1. Calcium leak through CNGC15 is predicted to positively feedback on DMI1, increasing its counter-balance flux, and by consequence the calcium release via CNGC15. (2) Holo-CaM2 binds CNGC15 and close CNGC15s whereas MCA8 pumps calcium back to the nuclear envelope lumen. (3) Holo-CaM2 binds upon release of calcium in the nucleoplasm. Each calcium release raises the nucleoplasmic calcium level above 700 nM. Holo-CaM2 binding to CNGC15s is transient and once holo-CaM2 is released from CNGC15, the cycle repeats itself as both ion channels are in an active state. (5) A yet unknown mechanism will stop the nuclear calcium oscillation. (B) $CaM2^{R91A}$ has higher affinity and association rate (K_{on}) than CaM2 to CNGC15, and thus outcompete CaM2 in *planta* leading to accelerated negative feedback (2), which is visualized by a reduction of the duration of the downward slope of each calcium spike and an increase of the calcium oscillation frequency. INE: Inner nuclear envelope. Figure created with BioRender.com

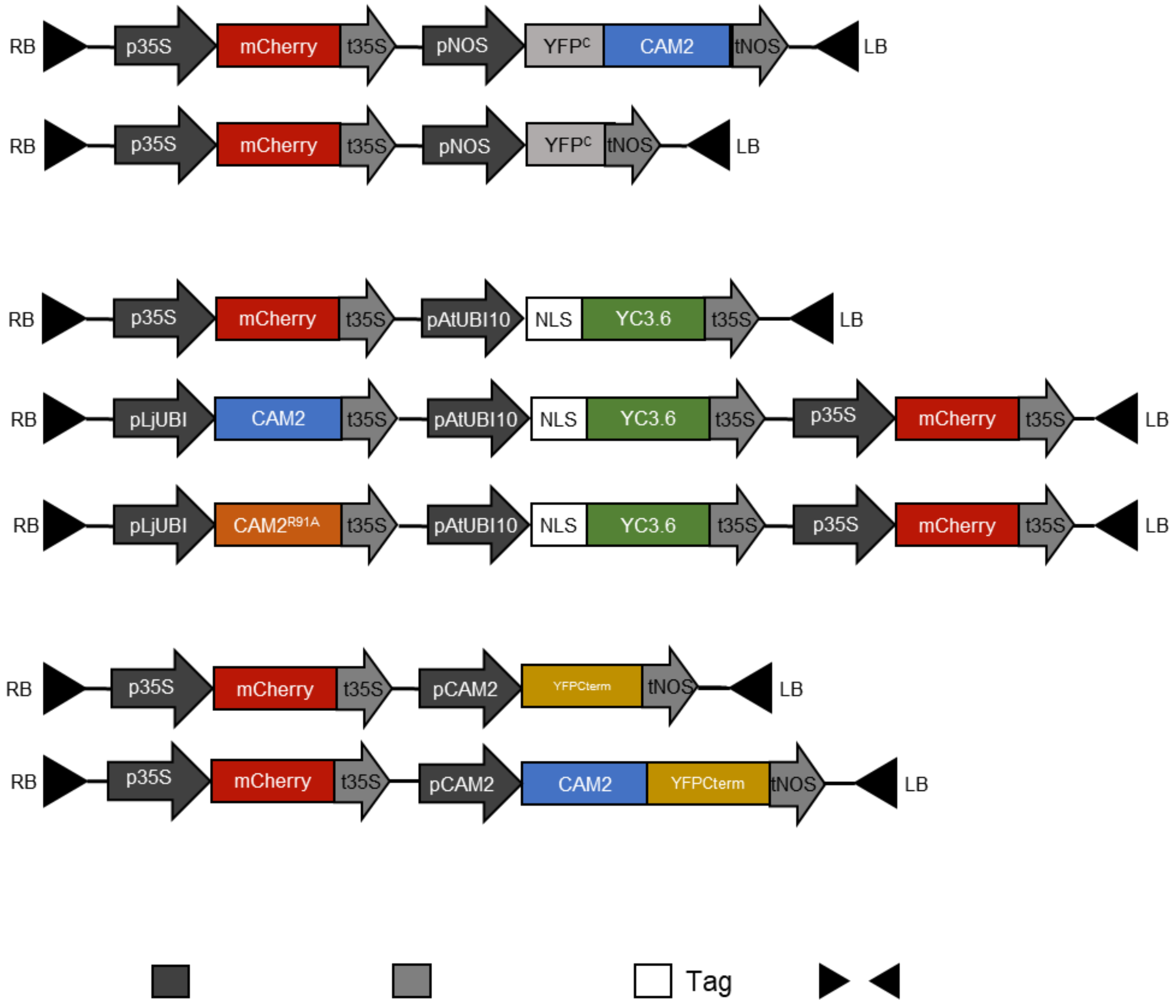

Fig. S16. GoldenGate constructs generated in this study.

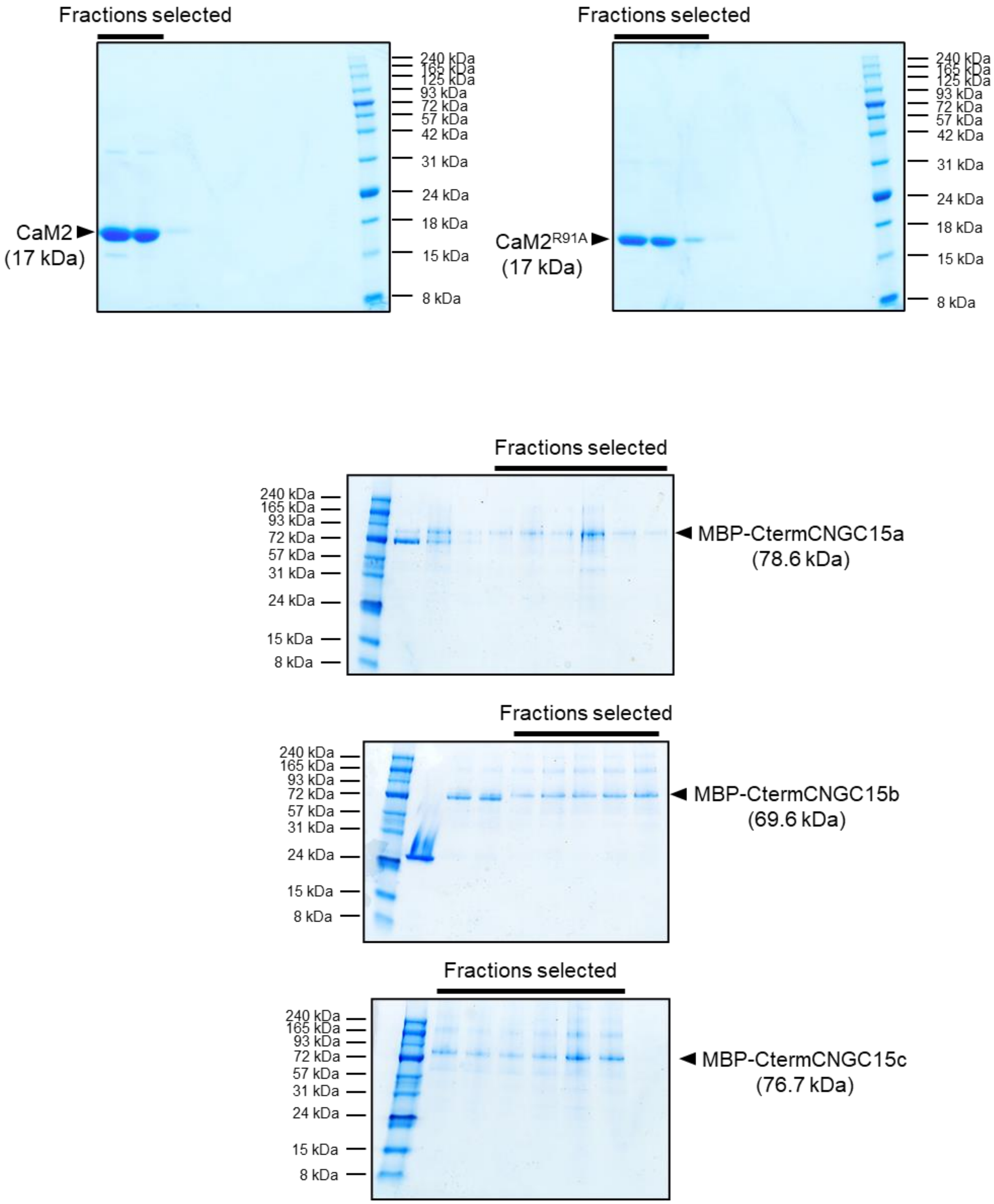

Fig. S17. Representative SDS-PAGE analyses of purified proteins.

CaMs (Halling et al., 2016)	MtCaM2			
Residue number in the interlobe linker with low frequency of conservation and exposed to the solvant (including Methionine as position 0)	Corresponding amino acid (inluding Methionine as position 1)	ΔΔG		$\Delta\Delta S_{vib}$
		DynaMut	ENCoM	ENCoM
66	P67A	0.733 (S)	0.004 (DS)	-0.005 (Decrease)
70	N71A	0.094 (S)	-0.115 (DS)	0.144 (Increase)
79	T80A	0.442 (S)	-0.177 (DS)	0.221 (Increase)
81	S82A	0.486 (S)	-0.098 (DS)	0.122 (Increase)
90	**R91A**	**-0.649 (DS)**	**-0.565 (DS)**	**0.323 (Increase)**

Table S1. Molecule stability and flexibility predicted from the DynaMut server upon the amino acid changes in CaM2.

ΔΔG : folding free energy (Kcal/mol); consensus calculated by DynaMut which includes comparison with ENCoM. $\Delta\Delta S_{vib}$: variation in entropy energy (Kcal/mol/K) between wild type and mutant which indicates an increase or decrease of flexibility. DS: Destabilizing, S: Stabilizing

Dataset S1 Table of the sequences used in the phylogenetic analysis.

Dataset S2 Table of the primers used in this study.

Dataset S3 Script file for calcium imaging analysis.

SI References

1. M. Fromont-Racine, J. C. Rain, P. Legrain, Toward a functional analysis of the yeast genome through exhaustive two-hybrid screens. *Nat Genet* **16**, 277-282 (1997).
2. R. D. Gietz, R. A. Woods, Genetic transformation of yeast. *Biotechniques* **30**, 816-820 (2001).
3. R. M. Horton, H. D. Hunt, S. N. Ho, J. K. Pullen, L. R. Pease, Engineering hybrid genes without the use of restriction enzymes: gene splicing by overlap extension. *Gene* **77**, 61-68 (1989).
4. D. M. Goodstein *et al.*, Phytozome: a comparative platform for green plant genomics. *Nucleic Acids Res* **40**, D1178-1186 (2012).
5. K. Katoh, D. M. Standley, MAFFT multiple sequence alignment software version 7: improvements in performance and usability. *Mol Biol Evol* **30**, 772-780 (2013).
6. L. T. Nguyen, H. A. Schmidt, A. von Haeseler, B. Q. Minh, IQ-TREE: a fast and effective stochastic algorithm for estimating maximum-likelihood phylogenies. *Mol Biol Evol* **32**, 268-274 (2015).
7. S. Kalyaanamoorthy, B. Q. Minh, T. K. F. Wong, A. von Haeseler, L. S. Jermiin, ModelFinder: fast model selection for accurate phylogenetic estimates. *Nat Methods* **14**, 587-589 (2017).
8. I. Letunic, P. Bork, Interactive Tree Of Life (iTOL) v4: recent updates and new developments. *Nucleic Acids Res* **47**, W256-W259 (2019).
9. K. Kakar *et al.*, A community resource for high-throughput quantitative RT-PCR analysis of transcription factor gene expression in Medicago truncatula. *Plant Methods* **4**, 18 (2008).
10. S. Pfeilmeier *et al.*, Expression of the Arabidopsis thaliana immune receptor EFR in Medicago truncatula reduces infection by a root pathogenic bacterium, but not nitrogen-fixing rhizobial symbiosis. *Plant Biotechnol J* **17**, 569-579 (2019).
11. C. Gehl, R. Waadt, J. Kudla, R. R. Mendel, R. Hansch, New GATEWAY vectors for high throughput analyses of protein-protein interactions by bimolecular fluorescence complementation. *Mol Plant* **2**, 1051-1058 (2009).
12. M. Karimi, D. Inze, A. Depicker, GATEWAY vectors for Agrobacterium-mediated plant transformation. *Trends in plant science* **7**, 193-195 (2002).
13. M. Charpentier, J. Sun, J. Wen, K. S. Mysore, G. E. Oldroyd, Abscisic acid promotion of arbuscular mycorrhizal colonization requires a component of the PROTEIN PHOSPHATASE 2A complex. *Plant Physiol* **166**, 2077-2090 (2014).
14. M. Groth *et al.*, NENA, a Lotus japonicus homolog of Sec13, is required for rhizodermal infection by arbuscular mycorrhiza fungi and rhizobia but dispensable for cortical endosymbiotic development. *The Plant cell* **22**, 2509-2526 (2010).
15. K. W. Bender *et al.*, Autophosphorylation-based Calcium (Ca(2+)) Sensitivity Priming and Ca(2+)/Calmodulin Inhibition of Arabidopsis thaliana Ca(2+)-dependent Protein Kinase 28 (CPK28). *The Journal of biological chemistry* **292**, 3988-4002 (2017).
16. S. Tyanova, T. Temu, J. Cox, The MaxQuant computational platform for mass spectrometry-based shotgun proteomics. *Nat Protoc* **11**, 2301-2319 (2016).
17. N. S. Berrow *et al.*, A versatile ligation-independent cloning method suitable for high-throughput expression screening applications. *Nucleic Acids Res* **35**, e45 (2007).

18. H. Tegel, S. Tourle, J. Ottosson, A. Persson, Increased levels of recombinant human proteins with the Escherichia coli strain Rosetta(DE3). *Protein Expr Purif* **69**, 159-167 (2010).
19. C. H. Rodrigues, D. E. Pires, D. B. Ascher, DynaMut: predicting the impact of mutations on protein conformation, flexibility and stability. *Nucleic Acids Res* **46**, W350-W355 (2018).
20. S. McNicholas, E. Potterton, K. S. Wilson, M. E. Noble, Presenting your structures: the CCP4mg molecular-graphics software. *Acta crystallographica. Section D, Biological crystallography* **67**, 386-394 (2011).
21. C. Engler, R. Gruetzner, R. Kandzia, S. Marillonnet, Golden gate shuffling: a one-pot DNA shuffling method based on type IIs restriction enzymes. *PLoS One* **4**, e5553 (2009).
22. D. W. Ehrhardt, E. M. Atkinson, S. R. Long, Depolarization of alfalfa root hair membrane potential by Rhizobium meliloti Nod factors. *Science* **256**, 998-1000 (1992).
23. G. Morieri *et al.*, Host-specific Nod-factors associated with Medicago truncatula nodule infection differentially induce calcium influx and calcium spiking in root hairs. *New Phytol* **200**, 656-662 (2013).
24. M. Giovanetti, B. Mosse, An evaluation of techniques for measuring vesicular-arbscule mycorrhizal infection in roots. *New Phytol* **84**, 489-500 (1980).
25. M. Charpentier *et al.*, Nuclear-localized cyclic nucleotide-gated channels mediate symbiotic calcium oscillations. *Science* **352**, 1102-1105 (2016).
